# *In Situ* Electron Paramagnetic Resonance Investigation of Isotope‐Selective Breathing in MIL‐53 During Dihydrogen Adsorption

**DOI:** 10.1002/chem.202500088

**Published:** 2025-01-31

**Authors:** Muhammad Fernadi Lukman, Sibo Chetry, Prantik Sarkar, Volodymyr Bon, Kavipriya Thangavel, Stefan Kaskel, Michael Hirscher, Harald Krautscheid, Andreas Pöppl

**Affiliations:** ^1^ Felix Bloch Institute for Solid State Physics Leipzig University Leipzig 04103 Germany; ^2^ Faculty of Chemistry and Mineralogy Institute for Inorganic Chemistry Leipzig University Leipzig 04103 Germany; ^3^ Max Planck Institute for Intelligent Systems Stuttgart 70569 Germany; ^4^ Chair of Inorganic Chemistry I Technical University Dresden Dresden D-01069 Germany; ^5^ National High Magnetic Field Laboratory Florida State University Tallahassee 32310 USA; ^6^ Institute of Separation Science and Technology Friedrich-Alexander-Universität Erlangen- Nürnberg (FAU) Erlangen 91058 Germany; ^7^ Advanced Institute for Materials Research (WPI-AIMR) Tohoku University Aoba-ku Sendai 980-8577 Japan

**Keywords:** *in situ*, EPR, dihydrogen, MIL-53, Breathing

## Abstract

The development of smart materials capable of separating dihydrogen isotopologues has risen recently. Among potential candidates, the flexible MIL‐53 (Al) has been gaining attention due to its structural flexibility providing the so‐called ‘’breathing mechanism’’ that can be useful to separate hydrogen isotopologues selectively. In the present work, an *in situ* continuous wave electron paramagnetic resonance investigation has been proven as a sensitive technique to follow the isotopologue‐selective adsorption‐desorption of dihydrogen species on the paramagnetic metal‐doped MIL‐53 (Al_0.99_Cr_0.01_) and MIL‐53 (Al_0.99_V_0.01_), respectively. The presence of paramagnetic spin probes such as Cr^3+^ and V^4+^ inside the MIL‐53 framework allows for monitoring the framework transition including the 2^nd^ transition step that selectively occurs at *p>*100 mbar when D_2_ gas is adsorbed on the pores at 23 K. Furthermore, investigation of D_2_ desorption from MIL‐53 (Al_0.99_V_0.01_) by temperature‐dependent hyperfine spectroscopy provides a more detailed analysis of the D_2_ desorption process on a microscopic scale with respect to the embedded spin probe.

## Graphical abstract

The *in situ* continuous wave EPR approach utilising Cr^3+^ and V^4+^ ions in the framework of MIL‐53 (Al) is successfully conducted to monitor the dihydrogen isotopologue‐selective breathing transition on the flexible MIL‐53 (Al) framework. In addition, the temperature‐dependent 3p ESEEM experiment can be implemented to sense the D_2_ from the perspective of the V^4+^ spin probe in the MIL‐53 (Al).

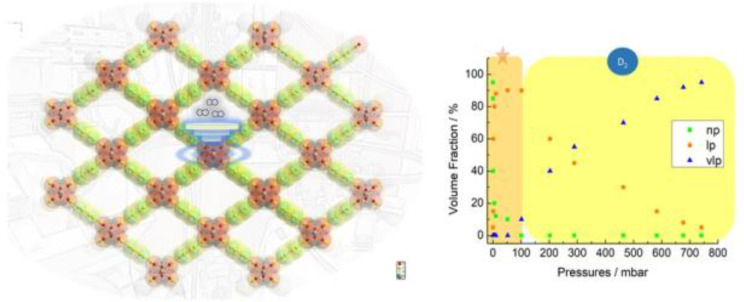

## Introduction

**Figure 1:**
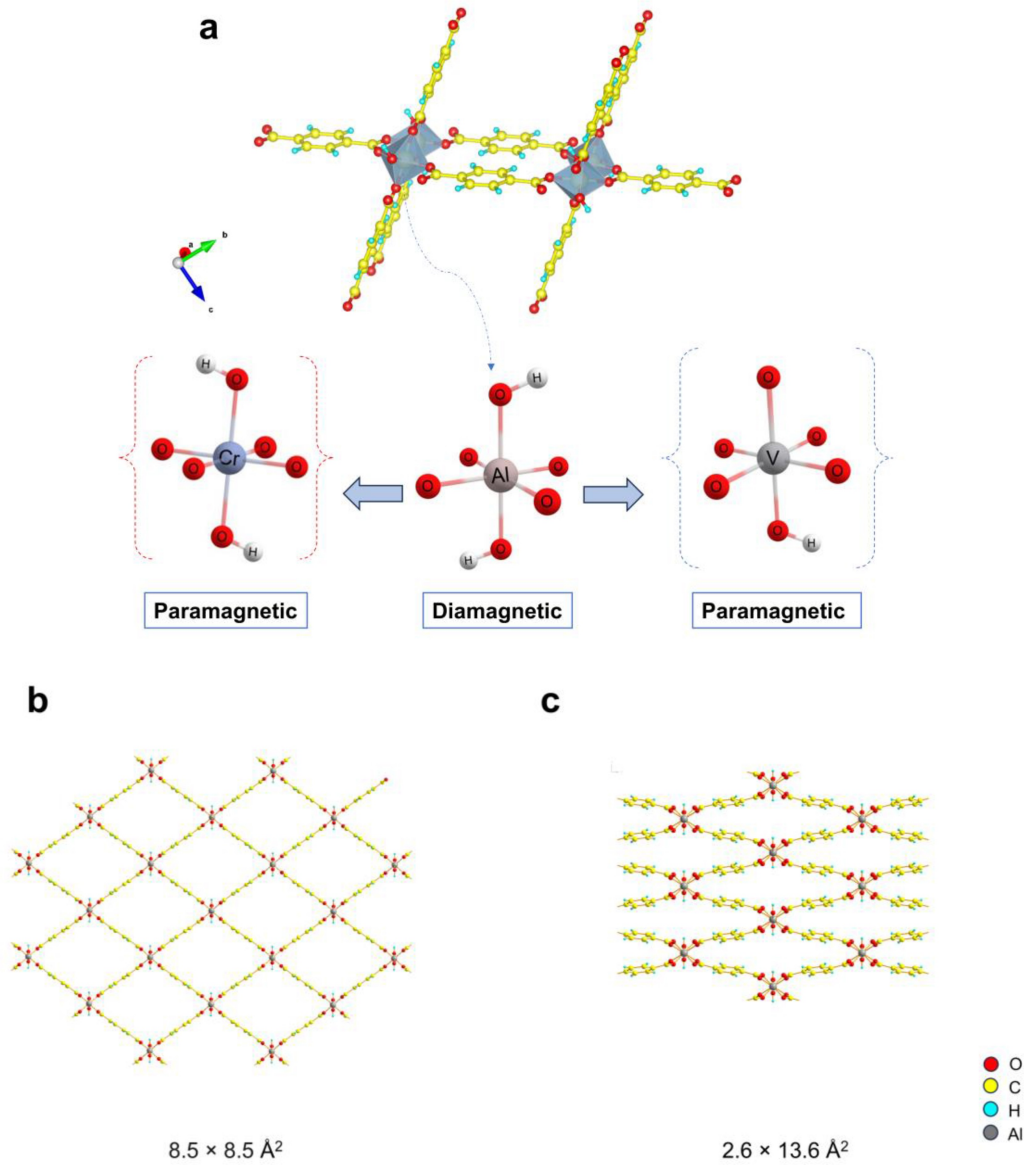
Schematic representation of the structure of MIL‐53 (Al) and its primary building units (AlO_6_ octahedra) (a) where the Al^3+^ site can be substituted either by V^4+^ or Cr^3+^ ions, respectively. The framework representations of (b) large pore phase, lp and (c) narrow pore phase, np and their pore channel dimensions are provided.

Deuterium and tritium are potentially useful as primary fuels for nuclear fusion reactors.^[1]^ When the demand for hydrogen isotopes rises in the future, it will require a more efficient technical procedure of the dihydrogen isotopologues separation,^[2]^ one possibility is to exploit the nanoconfinement effect^[3]^ that can be realized in nanoporous materials such as zeolites,^[4]^ metal‐organic frameworks (MOFs)^[5]^ or carbon‐based materials.^[6]^ The well‐known flexible MOFs such as MIL‐53 (Al)^[7]^ and DUT‐8 (Ni)^[8]^ are considered very attractive due to their switchability behaviour towards external stimuli like specific guest molecules, pressure or temperature.[^9^, ^10^, ^11^, ^12^, ^13^] MIL‐53 (Al) itself is composed of corner‐sharing octahedral secondary building units (Figure 1a) linked by the benzene dicarboxylate (bdc) ligands to generate a 3D structure with 1D lozenge‐like channels.^[7]^ Due to the structural flexibility, the overall framework geometry and pore size can adapt to the adsorption/desorption of guest molecules at a certain temperature. Two distinct pore phases have been well‐studied in the MIL‐53 (Al) framework[^7^, ^11^] and assigned to large pore (lp) and narrow pore (np) phases as illustrated in Figure 1b, c.

Recently, Kim *et al*.^[14]^ reported a D_2_‐selective breathing behaviour in the diamagnetic MIL‐53 (Al), capable of separating dihydrogen isotopologue mixtures. Based on *in situ* neutron powder diffraction (NPD) experiments and hysteresis profile from the volumetric sorption measurements of dihydrogen isotopologues, a ‘’very large pore (vlp)’’ phase in addition to the np and lp phases was proposed to explain the pore structure that allows the observed additional uptake of D_2_ starting from approximately 100 mbar (only at *T*<25 K) in comparison to the conventional adsorption behaviour of H_2_.^[14]^ From the perspective of detection tools, a combination of continuous wave (CW) and pulsed electron paramagnetic resonance (EPR) is a promising alternative to study the host‐guest interaction of MOFs and dihydrogen isotopologues when a proper spin probe is embedded in the MOF.[^15^, ^16^, ^17^] The CW‐EPR technique has been applied to confirm the isomorphous substitution of diamagnetic framework Al^3+^ ions by 1 % of paramagnetic Cr^3+^ ions in MIL‐53 (Al),^[18]^ which is deduced from the Cr^3+^ spin Hamiltonian parameters that are specific for each pore phase state of the flexible MOF via small changes of the coordination geometry of the Cr^3+^ spin probes on their octahedral framework site in MIL‐53 (Al). In addition, the CW EPR technique was implemented to monitor the np↔lp transitions in the temperature range of 5 K≤*T*≤400 K for MIL‐53 (Al_0.99_Cr_0.01_).^[18]^ The breathing transition between np and lp phases in response to CO_2_ adsorption was successfully monitored by *ex situ* ^[19]^ and *in situ* ^[20]^ CW EPR spectroscopy of Cr^3+^ for MIL‐53 (Al_0.99_Cr_0.01_). Separately, Nevjestic *et al*.^[21]^ have also demonstrated the possibility of incorporating V^3+^ ions into the MIL‐53 (Al). However, V^3+^ underwent oxidation during thermal activation to yield V^4+^ ions as vanadyl (VO^2+^). Nonetheless, the V^4+^ spin probe was still applicable as a sensor for gaseous O_2_ that adsorbed on MIL‐53 (Al).^[22]^

In this work, a highly sensitive *in situ* CW‐EPR approach is presented to directly monitor the selective breathing transitions of Cr^3+^‐ and V^4+^‐doped MIL‐53 (Al) in response to dihydrogen isotopologues adsorption and desorption. This allows us to explore the H_2_ and D_2_ physisorption process on a local microscopic scale as viewed from the framework metal ions. Electron spin echo envelope modulation (ESEEM) spectroscopy and thermal desorption spectroscopy (TDS) were complementarily deployed to study the desorption process of dihydrogen isotopologues (H_2_ and D_2_) on Cr^3+^‐ and V^4+^‐doped MIL‐53 (Al). Here, the application of ESEEM spectroscopy offers the opportunity to monitor directly the presence of adsorbed D_2_ molecules in the pores of the V^4+^‐doped MIL‐53 (Al) and to observe their mobilization in the advent of the desorption process. The experiments are separately performed for H_2_ and D_2_ adsorption in this work to evaluate the potential of CW and pulsed EPR spectroscopy in the field of dihydrogen isotopologues detection but might be applied to explore spectroscopically on a microscopic scale also separation processes in the future.

## Results & Discussion

### *In Situ* CW‐EPR: Dihydrogen Isotopologues Adsorption on Cr^3+^‐Doped MIL‐53

**Figure 2:**
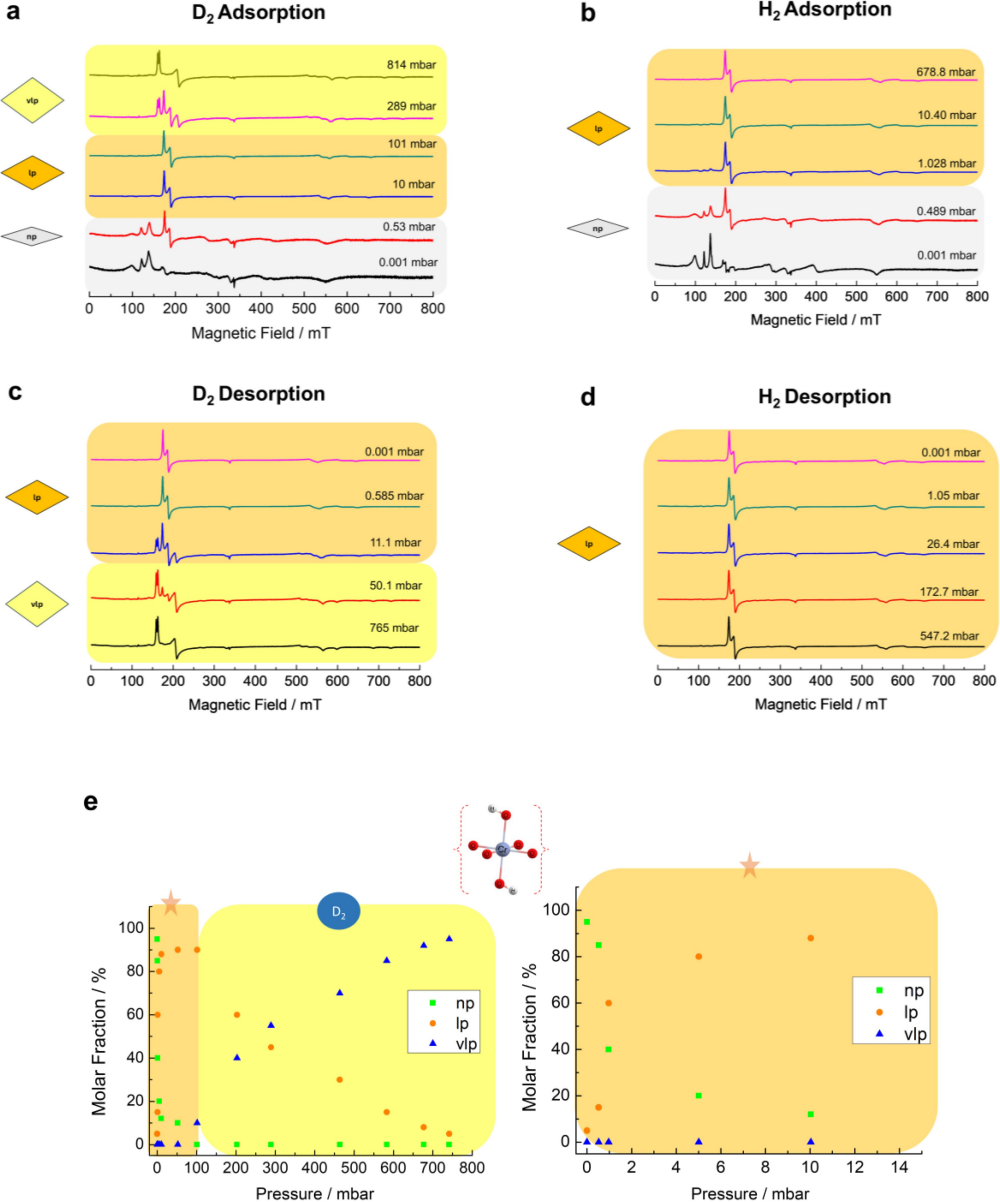
(a) Selected *in situ* CW‐EPR spectra of Cr^3+^ species in the MIL‐53(Al_0.99_Cr_0.01_) while adsorbing D_2_ gas from *p*=0.001 mbar to *p*=814 mbar measured at 23 K and (b) during adsorption of H_2_ gas from *p*=0.001 mbar to *p*=678.8 mbar measured at 20 K. (c) represents the desorption branch for D_2_ gas from *p*=765 mbar to *p*=0.001 mbar and (d) represents the desorption branch for H_2_ gas from *p*=547.2 mbar to *p*=0.001 mbar. (e) the percentage of the molar fraction of np, lp and vlp species during *in situ* adsorption of D_2_ at 23 K. For clarity, the low‐pressure region (*p*<15 mbar) is enlarged on the right side. A complete set of EPR spectra is provided in SI, Figure S19.

**Figure 3:**
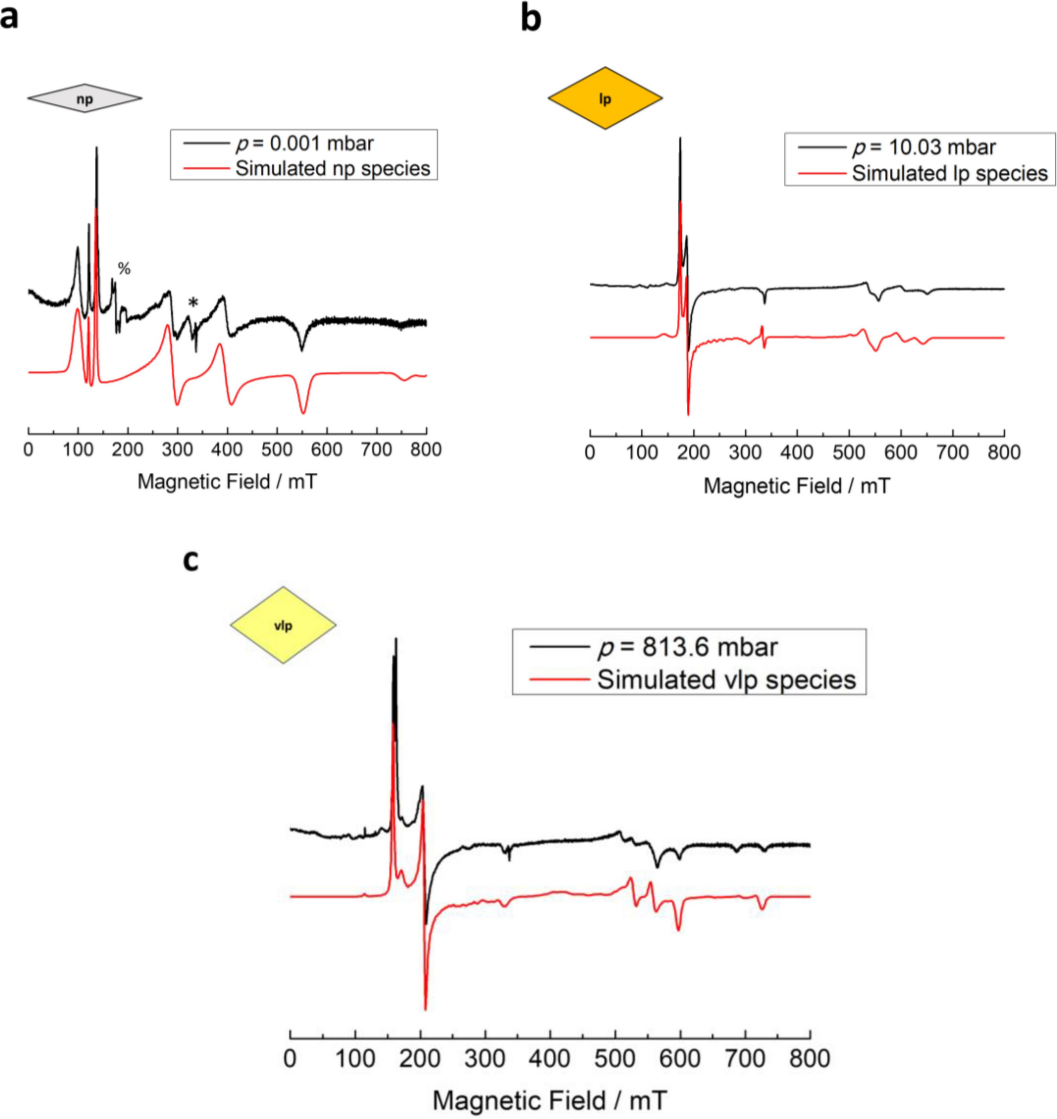
The spectral simulation (red) corresponds to the Cr^3+^ speciation in three distinct pore phases to describe the experimental spectra (black) of D_2_ adsorbed on MIL‐53 (Al_0.99_Cr_0.01_) at (a) 0.001 mbar (np phase), (b) 10.03 mbar (lp phase), and (c) 813.6 mbar (vlp phase) recorded at T=23 K. Signals marked by % and *are attributed to the residual lp phase and organic radical impurities, respectively.

In this section, the adsorption of dihydrogen isotopologues on flexible MIL‐53 (Al_0.99_Cr_0.01_) is discussed where at least two pore phase transitions have been identified for D_2_ adsorption at 23 K: np to lp and lp to vlp states according to the volumetric sorption data from Kim *et al*.^[14]^ EPR spin Hamiltonian parameter and contribution of the EPR intensities of pore phases (*I*_np,lp,vlp_) were deduced from the simulation of the EPR spectra (see SI section 1.2 for the details). Before D_2_ gas loading, EPR spectra recorded at the activated state (*p*=0.001 mbar at 23 K) indicate the Cr^3+^ ions in the np phase with zero‐field splitting (zfs) parameters *D*=6.84 GHz and *E*=1.64 GHz, in line to the previously published EPR results,[^18^, ^19^] are dominant contributors to the spectrum with *I*_np_ *=*0.94 (94 %) (see Figure 2a and 3a for the CW EPR spectra recorded *in situ* and Table 1 for the complete spin Hamiltonian parameters). The rest of the signal can be assigned to residual Cr^3+^ species in the lp phase with *I*_lp_ *=*0.05 (5 %) and the minor impurity related to organic radicals (<1 %). When the pressure of D_2_ loading is raised to 10 mbar, the lp species with zfs parameters *D*=8.65 GHz and *E*=0.19 GHz overtake the np species as the major contributor with approximately 60 %. This implies the phase transition from np to lp phases as the D_2_ gas pressure builds up. The lp species possesses a slightly larger *D* and significantly lower *E/D* ratio if compared to the np species (Table 1 and Figure 3b for the spectral simulations). The results are comparable with pervious Cr^3+^ data for the np and lp phase in MIL‐53 (Al_0.99_Cr_0.01_) obtained by Mendt et al.,^[18]^ where the np→lp phase transition was induced either by lowering the temperature^[18]^ or by adsorption of CO_2_.^[18]^ It can be inferred from those EPR parameters that the chromium octahedra in the lp phase exhibit a distinctly smaller rhombic distortion in comparison with the np phase due to minimum spatial constraints from the linkers as previously reported by Mendt and co‐workers,^[18]^ reflected from the significant decrease (ca. 10 fold reduction) of the *E*/*D* ratio (Table 1). Moreover, the increment of the axial zfs parameter, *D*, in the context of the np to lp transition is probably correlated to an elongation of the axial Cr−O bond in the Cr octahedron. This indication is supported by the structural data provided in Table S2 where the axial distortion parameter *Δ*_ax_=I *R*_ax_–*R*_eq_ I/*R*_ax_ with the axial and equatorial Al−O bond lengths *R*_ax_ and *R*_eq_, respectively, is increasing and the <O−Al−O and <OH−Al‐O are both approaching 90° for the lp phase.

**Table 1:** Set of spin Hamiltonian parameters for Cr^3+^ ions in the MIL‐53 (Al_0.99_Cr_0.01_) framework upon *in situ* adsorption experiments. *ΔD* and *ΔE* are the axial and rhombic zero field splitting strain parameters, respectively. Here *g* refers to the *g*_iso_ value.

Species	*g*	*D*/GHz	*E*/GHz	*E/D*	Δ*D/*GHz	Δ*E/*GHz
np	1.977(5)	6.84(2)	1.64(1)	0.239(2)	0.55(10)	0.15(10)
lp	1.975(5)	8.65(4)	0.19(1)	0.022(1)	0.48(10)	0.08(3)
vlp	1.970(5)	9.10(4)	0.80(1)	0.088(1)	0.40(10)	0.01(3)

The np species contribution to the EPR spectrum diminishes as the D_2_ pressure increases to 100 mbar. At 100 mbar, the lp species rises to 85 %. Interestingly, another species becomes observable at this gas pressure as well. This new species gradually dominates as a major contributor to the EPR spectra at the expense of lp species until reaching its maximum when recorded at much higher D_2_ pressure (ca. 814 mbar). We assign this species to Cr^3+^ ions located in the vlp phase which is formed at these D_2_ gas pressure according to previous NPD analysis and volumetric sorption experiments.^[14]^ The spectral simulation in Figure 3c suggests that the vlp species exhibit slightly higher *D* and *E* values than the lp species. This vlp species is characterized by the zero‐field splitting parameter *D*=9.10 GHz and *E*=0.80 GHz. Concerning the lp to vlp transition, the Cr^3+^ ions in the vlp phase exhibit a larger axial zfs parameter *D* and a slightly enhanced *E*/*D* ratio than in the lp phase which signifies a slightly lower symmetry of the Cr^3+^ octahedron sites of vlp phase. In accordance with the trend of the Cr^3+^ zfs parameters, the axial distortion parameter *Δ*_ax_ increases from 0.022 to 0.063 (see Table S2) and the Al−O bond angles (<OH−Al−O and <O−Al−O) deviate significantly from 90° even though the changes in the lp to vlp case is not as large as for the np to lp transition. A breathing diagram for D_2_ gas adsorption at 23 K can be derived from *in situ* CW EPR spectra and is presented in Figure 2e.

In contrast to D_2_ adsorption over MIL‐53 (Al_0.99_Cr_0.01_), there is only one phase transition stage (np to lp phase) that occurs for the H_2_ adsorption even when the pressure reaches *p*=678.8 mbar at 20 K (Figure 2b). *In situ* CW‐EPR data for both H_2_ and D_2_ gases at 40 K (Figure S3) have exclusively implied that the vlp species is only present below 25 K within the pressure limit of our current *in situ* setup (1 bar).

The difference between the CW‐EPR spectral evolution in response to H_2_ and D_2_ desorption was also detectable. *In situ* CW‐EPR data for the desorption branch are provided in Figure 2c, d showing that the vlp species can be transformed back to the lp species only below 10 mbar whereas the lp phase stays during H_2_ gas desorption at 23 K. Consistently, these findings are in close agreement with the breathing for the pure MIL‐53 (Al) system^[14]^ under similar adsorption‐desorption conditions. Therefore, the results of *in situ* EPR investigations of the local Cr^3+^ spin probes serve as further direct evidence of the isotope‐selective phase transitions effect toward D_2_ gas as opposed to H_2_ gas in MIL‐53 (Al_0.99_Cr_0.01_) and provide information about the changes of the local structure of CrO_6_ octahedra related to both D_2_ or H_2_ gas adsorption‐triggered breathing transition.

### *In Situ* CW‐EPR : Dihydrogen Isotopologues Adsorption on V^4+^ Doped MIL‐53

Paramagnetic vanadium ions incorporated at the Al^3+^ framework sites may also serve as an alternative spin probe to explore dihydrogen adsorption‐triggered breathing in MIL‐53 (Al). As previously reported by Kozachuk *et al*.,^[23]^ in our X band EPR investigations, we also identified V^4+^ (VO^2+^, 3d^1^, S=1/2) ions, even though MIL‐53 (Al_0.99_V_0.01_) was initially doped by V^3+^ ions (3d^2^, *S*=0, 1). In addition, V^4+^ ions exhibit distinct *g* tensor and ^51^V hyperfine (HF) tensor parameters for the np and lp phase as recently reported.^[23]^

**Figure 4:**
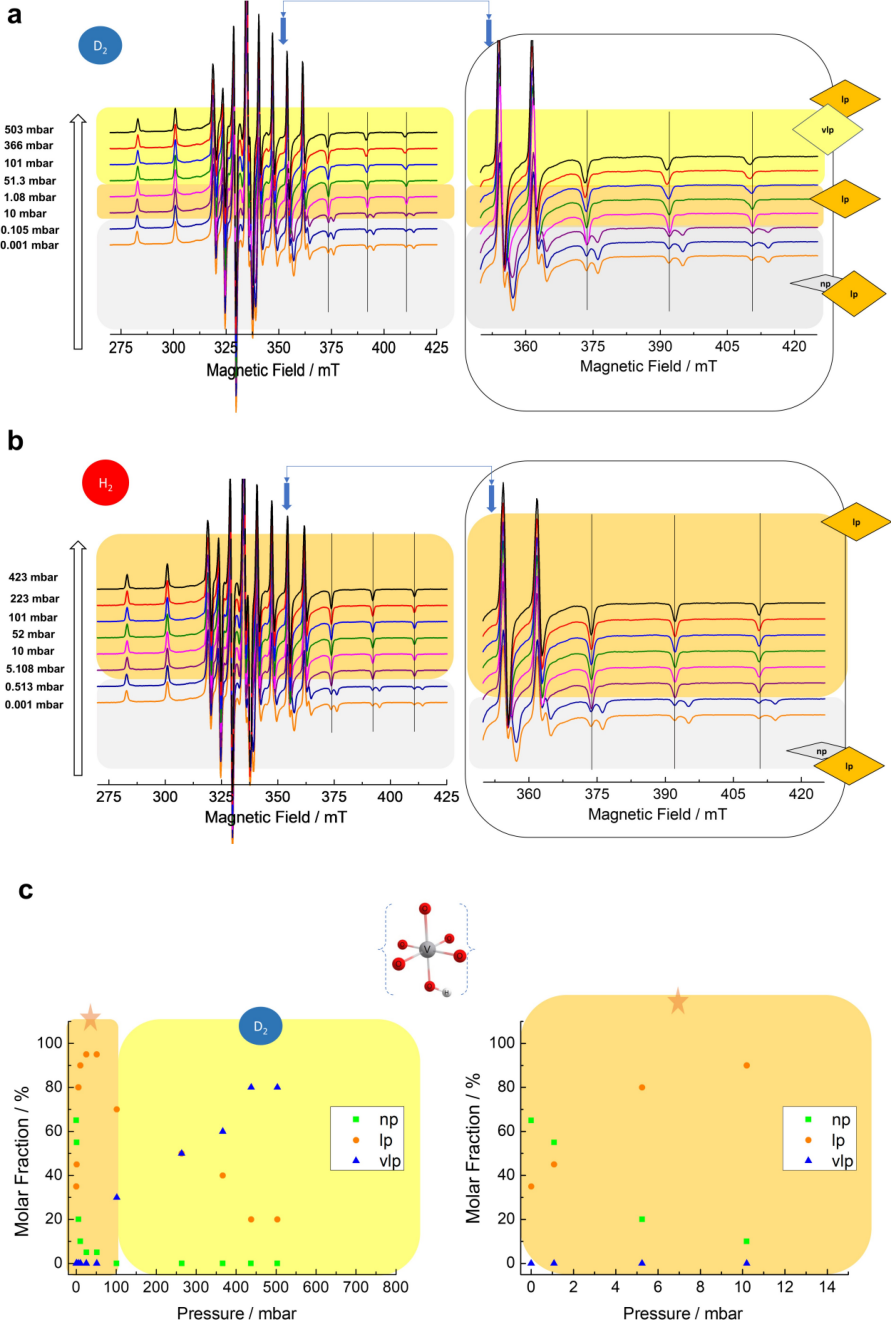
(a) *In situ* CW‐EPR spectra of V^4+^ species in MIL‐53(Al_0.99_V_0.01_) while adsorbing D_2_ gas from *p*=0.001 mbar to *p*=503 mbar measured at 21 K and (b) during adsorption of H_2_ gas from *p*=0.001 mbar to *p*=423 mbar measured at 20 K. (c) the molar fraction of each pore phase species as determined from simulations of the V^4+^ *in situ* EPR spectra during adsorption of D_2_ over MIL‐53 (Al_0.99_V_0.01_) (left panel) from *p*=0.001 mbar to *p*=503 mbar at 21 K and (right panel) expanded low‐pressure region (marked as a star) *p*<15 mbar.

**Figure 5:**
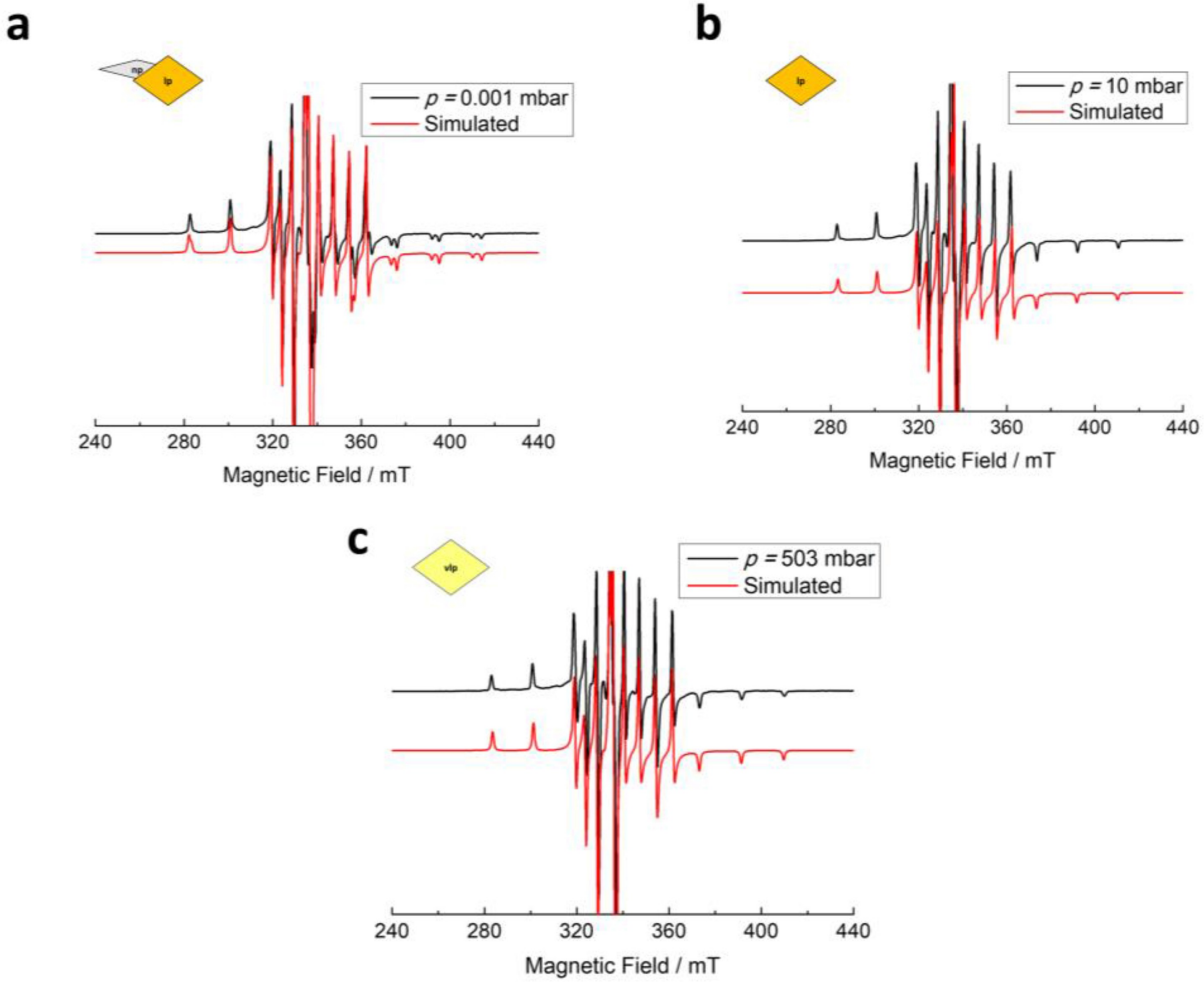
The spectral simulation (red) corresponds to the V^4+^ speciation at three distinct pore phases to describe the experimental spectra (black) of D_2_ adsorbed on MIL‐53 (Al_0.99_V_0.01_) at (a) 0.001 mbar, (b) 10 mbar and (c) 503 mbar (recorded at T=21 K).

The EPR spectrum of the activated state of MIL‐53 (Al_0.99_V_0.01_) (*p=*0.001 mbar in Figure 4a) exhibits the superposition of two distinct V^4+^ species, which can be assigned again to the np and lp phase.^[23]^ A similar quantitative approach using eq. (3) from SI section 1.2 is conducted to account for the contribution of each pore phase species in the EPR spectra. The V^4+^ np species with axially symmetric *g* and ^51^V HF parameters of *g*_zz_=1.930 and $A_{zz}^{V}$ =510 MHz as deduced from spectral simulations (see Figure 5a) has a dominant contribution (65 %) in comparison to the lp species having *g*_zz_=1.938 and $A_{zz}^{V}$ =495 MHz (35 %). If one compares the np species contribution for the activated state of MIL‐53 (Al_0.99_Cr_0.01_) which contains Cr^3+^ ions (94 %) versus MIL‐53 (Al_0.99_V_0.01_), containing V^4+^ ions, (65 %), it can be seen that MIL‐53 (Al_0.99_V_0.01_) exhibit a lower np phase proportion than in MIL‐53 (Al_0.99_Cr_0.01_) presumably due to local structural distortions of the framework when V^3+^ is oxidized to V^4+^ ions. Furthermore, the crystal size also plays an important role in rationalizing the discrepancy of pore phase proportion^[24]^ in the two samples as can be inferred from the SEM images (Figure S17) that MIL‐53 (Al_0.99_V_0.01_) has a smaller crystal size plus no observable hysteresis in the N_2_ sorption data (Figure S13).

The phase analysis for V^4+^ and Cr^3+^ doped MIL‐53 (Al) has been obtained by variable temperature PXRD experiments and described in SI section 1.1. Kozachuk *et al*.^[23]^ suggest that the V^4+^ site deviates from octahedral to a pseudo‐octahedral or a square‐pyramidal configuration. At a total vanadium concentration >27 % they found a purely rigid framework with 99 % lp phase triggered by the likewise enhanced V^4+^ concentration and subsequently a larger number of distorted metal ion sites in the framework, preventing the lp→np phase transformation. Therefore, we assign the V^4+^ lp species in MIL‐53 (Al_0.99_V_0.01_) to a fraction of crystallites (35 %) with enhanced V^4+^ concentration possessing the lp phase, where the lp→np phase transformation is suppressed. The remaining V^4+^ np species belong to the larger molar fraction of crystallites (65 %) with a lower V^4+^ concentration which still undergoes the breathing transition typical for MIL‐53 (Al). Interestingly, the V^4+^ spin probes are still a sensitive observer for those flexible crystallites towards adsorption and desorption of dihydrogen isotopologues as depicted in Figure 4a, b (for D_2_ and H_2_ adsorption) and Figure S4 (for D_2_ and H_2_ desorption).

The lp species gradually becomes the major species from *p*=10 mbar to *p*=100 mbar (95 %) as depicted in Figure 4a. Moreover, Figure 4c displays the breathing diagram deduced from the *in situ* CW‐EPR spectra due to D_2_ adsorption. The EPR spectral patterns of both D_2_ and H_2_ adsorption branches are similar within this pressure range (*i. e*. 0.001–100 mbar (Figure 4a, b). However, rather drastic spectral changes were only observable for D_2_ adsorption after reaching 100 mbar with the apparent inhomogeneous broadening and field shifts of the ^51^V HF signals, especially pronounced in the magnetic field range of 360–420 mT as a result of further V^4+^ species occurrence which is superimposed with the signals of the lp species. According to the spectral simulation, this species exhibits *g*_zz_=1.939 and $A_{zz}^{V}$ =489 MHz (Table 2 for the spin Hamiltonian parameters and Figure 5c for the simulation). We correlate this species with the vlp phase based on our previous assignments of the pore phases in MIL‐53 (Al_0.99_Cr_0.01_). Both np→lp and lp→vlp transitions result in local geometry changes of the local vanadium coordination and thus alter the *g*‐tensor and ^51^V HF parameters. In order to rationalize the trend of *A*_zz_ increase and *g*_zz_ decrease, the evolution of V=O bonding properties is expected to play an important role. Carl and co‐workers^[25]^ explained the *A*_zz_ decrease and *g*_zz_ increase trends in the case of V^4+^ exchanged zeolites with the V=O bond length increase in the V^4+^ octahedron. Again the increase of the axial distortion parameter *Δ*_ax_ from np to lp is useful to interpret a significant reduction of the ^51^ V HF coupling and at the same time, a significant increase in the *g*_zz_ parameter. Fundamentally, according to the formula of A_zz_ and *g*_zz_ provided by Kivelson and Lee,^[26]^ the increase of *g*_zz_ might be roughly interpreted as a lowering of the energy difference between d_xy_ and d_x2‐y2_ orbitals of the V^4+^ ions. In addition, a relatively smaller increase of *g*_zz_ and A_zz_ decrease from lp to vlp is also consistent with the general trend of the Al−O bond properties given in Table S2. Again, the phase transition diagram for the adsorption of D_2_ and H_2_ (Figure 4) can be deduced from the weighting factors of the V^4+^ subspectra determined from EPR spectral simulations.

**Table 2:** Set of spin Hamiltonian parameters for V^4+^ ion in the MIL‐53 (Al_0.99_V_0.01_) framework upon *in situ* adsorption experiments. Axially symmetric *g* and A tensors are assumed in the simulations.

Species	*g*_xx,yy_	*g_zz_*	*A*_xx,yy/_MHz	*A*_zz*/*_MHz
np	1.966(2)	1.930(2)	167(4)	510(4)
lp	1.966(2)	1.938(2)	167(3)	495(3)
vlp	1.969(2)	1.939(2)	165(4)	489(4)

Furthermore, during the desorption of the D_2_ gas (Figure S4), the V^4+^ *in situ* EPR experiments revealed a vlp→lp phase transformation when the pressure is reduced below 40 mbar. Separately, no phase transition has been observed in the V^4+^ *in situ* EPR experiments during H_2_ gas desorption.

### Thermal Desorption Spectroscopy (TDS)

**Figure 6:**
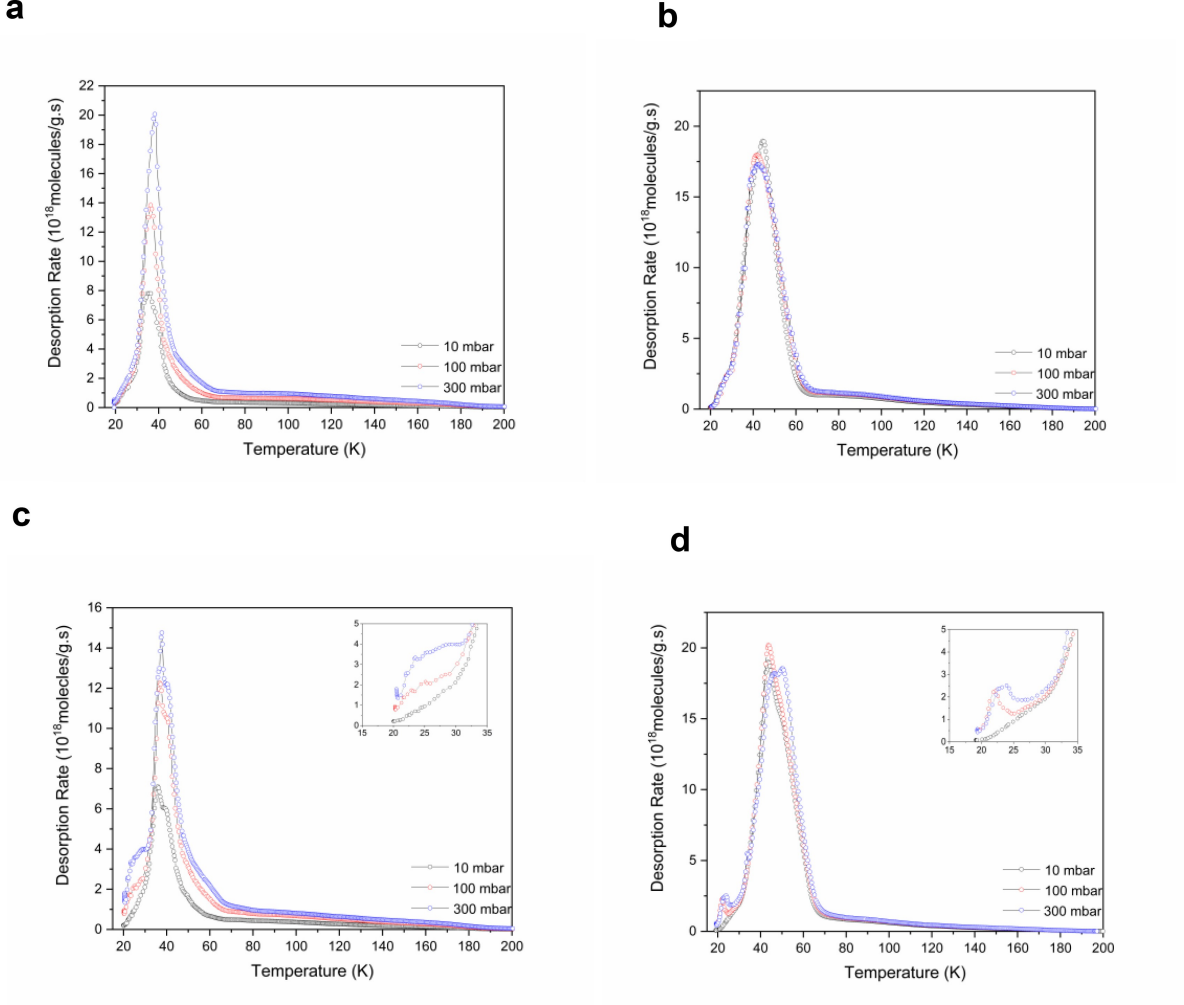
TDS results of MIL‐53(Al_0.99_Cr_0.01_) for (a) pure H_2_ gas, (c) pure D_2_ gas, and MIL‐53(Al_0.99_V_0.01_) for (b) pure H_2_ gas, and (d) pure D_2_ gas, exposed at 20 K. Onset graphs indicate an extra small peak for D_2_ at 300 mbar in the temperature range of 22–25 K.

Advanced cryogenic TDS measurements were conducted to identify different adsorption sites. After exposure to pure H_2_ or D_2_ at 20 K and 23 K, respectively, at different exposure pressure, the desorption of dihydrogen isotopologues was detected using a quadrupole mass spectrometer.^[27]^ For exposure at 10 mbar, one desorption peak is observed at a maximum temperature of 36 K and 44 K for MIL‐53 (Al_0.99_Cr_0.01_) and MIL‐53 (Al_0.99_V_0.01_), respectively (Figure 6). TDS results from Oh *et al*.^[28]^ with undoped MIL‐53 (Al) showed a single broad peak at about 40 K. This single desorption peak results from the one‐dimensional channel flexibility, even though at least four energetically different sorption sites^[14]^ have been observed by NPD experiments. At higher exposure pressure (100 and 300 mbar) an additional low‐temperature desorption maximum at 23 K appears only for D_2_ but is not observable for H_2_ exposure. This new desorption maximum for D_2_ occurs in the specific pressure and temperature range where the EPR experiments observe the vlp phase. Furthermore, it appears in the same range as the second transition step in the D_2_ adsorption isotherm previously assigned to the vlp phase^[28]^. Small residual desorption traces of dihydrogen isotopologues at a higher temperature (up to 140 K) can be explained by the molecular trapping after lp to np phase transition as Pollock *et al*. suggested in their NPD experiments.^[29]^

### Pulsed EPR Approach for D_2_ Adsorption‐Desorption on V^4+^ Doped MIL‐53

3p ESEEM and hyperfine sublevel correlation (HYSCORE) spectroscopy were implemented to explore the adsorption and desorption of D_2_ in the pores of MIL‐53 (Al_0.99_V_0.01_) on a microscopic scale_,_ viewed from the V^4+^ spin probes. These techniques allow the detection of the adsorbed D_2_ molecules in the vicinity of the spin probes and further direct observation of the desorption process of the D_2_ molecules in the proximity of the V^4+^ ions up to *T*≤40 K. Please note that the adsorption‐desorption of D_2_ here is obtained in a closed‐loop system where it is possible to maintain the total number of D_2_ molecules in equilibrium between the pores and the gas phase (*i. e*. without pumping out the gas from the sample tube). The V^4+^ with *S*=1/2 system was chosen for this approach instead of the Cr^3+^ spin probe (*S*=3/2) considering longer electron spin relaxation times and simpler pulsed EPR spectral interpretation for the V^4+^ ions at the X‐band frequency.

The field‐swept electron spin echo (FS ESE) spectra of D_2_ adsorbed on MIL‐53 (Al_0.99_V_0.01_) and the activated state are given in Figure S5. The *ex‐situ* adsorption of 133 mbar D_2_ at 77 K is within the adequate pressure regime to induce a phase transition from the mixtures of np and lp phases toward the lp phase as can be identified nicely from the FS ESE. The corresponding first‐derivative of the FS ESE spectra signifies the first breathing effect and the simulated parameters for those FS ESE spectra are identical to the previously discussed *in situ* CW‐EPR speciation analysis at the activated state (mixtures of np and lp species) and D_2_ adsorbed (predominantly lp species) states, respectively (see Table 2).

A comparison of the time domain 3p ESEEM spectra between MIL‐53 (Al_0.99_V_0.01_) adsorbed with D_2_ and its activated state reveals the presence of D_2_ in the proximity of intraframework V^4+^ sites (Figure S6). The 3p ESEEM spectra of the activated state recorded with a pulse delay *τ*=134 ns show typical ^27^Al modulation with a period of 260 ns (corresponding to the ^27^Al Larmor frequency *ν*_Al_=3.85 MHz) due to weak HF interactions with surrounding octahedral Al framework sites. On the other hand, a 3p ESEEM spectrum recorded with *τ*=218 ns shows a characteristic modulation of ^1^H with a period of about 68 ns (corresponding to *ν*_H_=14.74 MHz) which is indicative of weakly coupled ^1^H nuclei of the organic linkers and distant μ_2_‐OH bridging groups. On the other hand, ^27^Al modulation is suppressed in this specific *τ* value. Due to the blind spot effect,^[30]^ the ^1^H modulation vanishes at the 3p ESEEM spectra recorded with *τ*=134 ns. Successful detection of the adsorbed D_2_ gas is marked by a clear appearance of an envelope modulation with a period of 450 ns which correlates to the deuterium Larmor frequency *ν*_D_=2.21 MHz. HYSCORE^[31]^ spectra were recorded before and after the *ex‐situ* adsorption of D_2_ gas on the MIL‐53 (Al_0.99_V_0.01_) sample (Figure S7). The results discussed in SI section 1.3 indicate an almost zero isotropic HF coupling and a dipolar HF coupling parameter *T*=0.1 MHz of the adsorbed D_2_ molecules. Using a simple point‐dipole approximation^[32]^ the latter suggests a V^4+^‐D_2_ distance larger than approximately 0.45 nm and provides additional proof of weakly coupled D_2_ adsorption on the pores, specifically in the vicinity of the V^4+^ spin probe. This result agrees with the Al^3+^ ‐D_2_ distance obtained by the neutron scattering data.^[29]^

**Figure 7:**
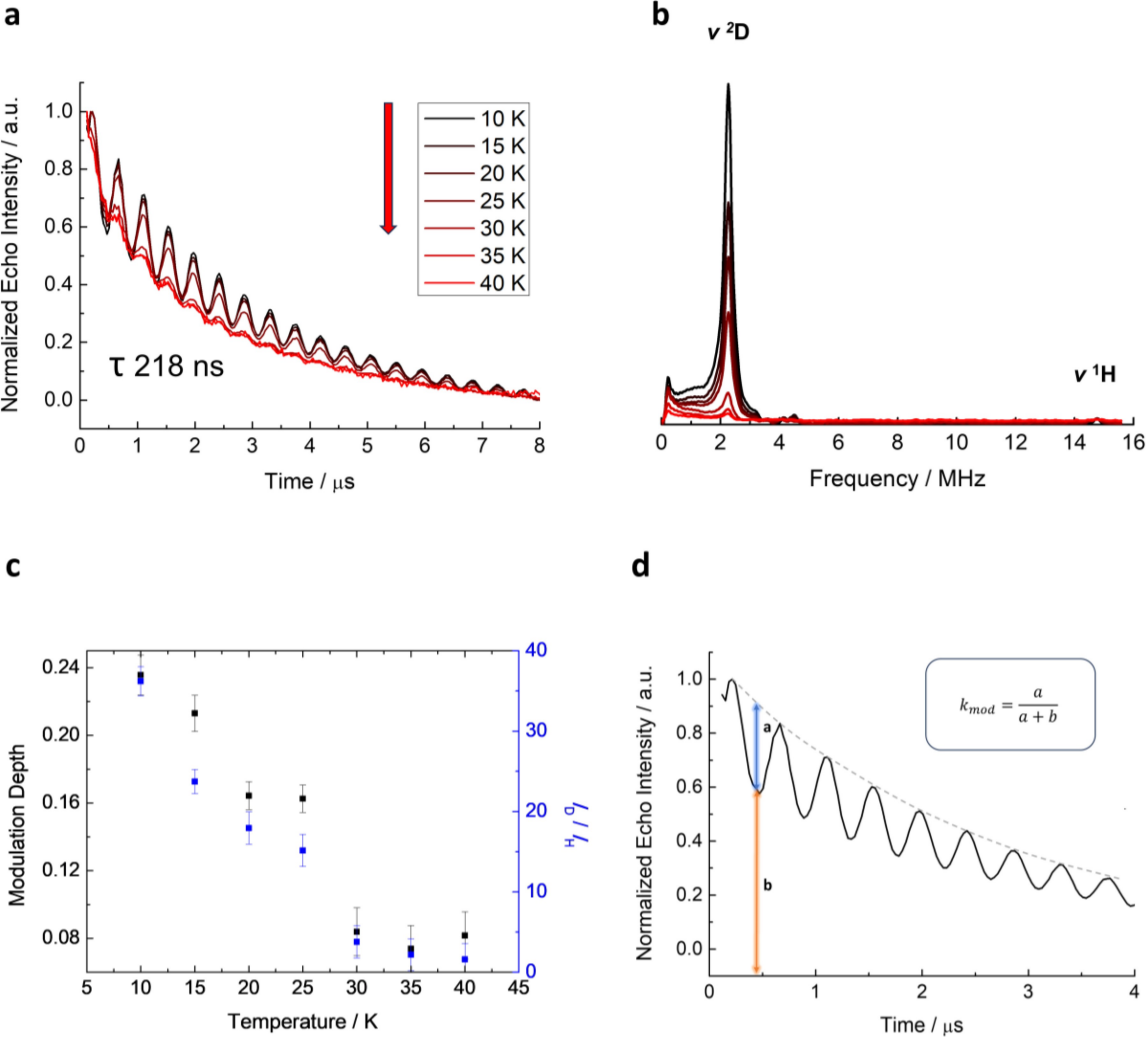
Temperature‐dependent 3p ESEEM of D_2_ desorption on MIL‐53(Al_0.99_V_0.01_) as (a) time domain and (b) frequency domain plot. Additionally, (c) the modulation depth *k*_mod_ and intensity ratio of *I*_D_/*I*_H_ are plotted as a function of temperature to illustrate the desorption of D_2_ gas. In addition, (d) the *k*_mod_ definition for the time domain dataset is also provided.

The desorption process of the D_2_ gas is monitored locally by temperature‐dependent 3p ESEEM spectroscopy as illustrated in Figure 7. As the temperature rises stepwise from 10 K to 40 K, a gradual decrease in the deuterium modulation depth in the 3p ESEEM time domain traces has been observed (Figure 7a) which can be quantified by the temperature dependence of the modulation depth parameter *k*_mod_ (for the definition of *k*_mod_ see Figure 7d). Here, *k*_mod_ depends on the spin Hamiltonian parameters of the interacting deuterium nuclei, thus their distances from the paramagnetic ion, and their number.[^15^, ^33^, ^34^] In the present case of weakly interacting nuclei their number can be considered the major parameter influencing *k*_mod_. Therefore we may refer to *k*_mod_ as an approximate measure for the D_2_ density^[35]^ in the vicinity of the V^4+^ probe.

The same holds for the intensities *I*_mod_ of the signals of the weakly coupled deuterium nuclei at *ν*_D_=2.21 MHz in the 3p ESEEM frequency domain spectra (Figure 7b) They show likewise a decrease of the intensity of the deuterium signal of the adsorbed D_2_ molecules at *ν*_D_=2.21 MHz with rising temperature. Notably, the frequency domain ESEEM spectra were normalized to the ^1^H signal at the proton free Lamor frequency *ν*_H_=14.74 MHz of the framework protons to account for the decrease in electron spin echo signal intensity with rising temperature due to enhanced spin‐lattice relaxation. It is justified to assume that this ^1^H ESEEM signal from the weakly coupled framework protons is not affected by the D_2_ desorption process, as the pulsed EPR experiments have solely performed in the lp phase. In this case the V^4+^‐^1^H distances are fixed and consequently the HF parameters, which determine exclusively together with the constant number of interacting framework protons the width and intensity of the proton signal at the free Lamor frequency, do not change (Figure S18). The intensities *I*_mod_ of the deuterium signals normalized in this way are likewise plotted in Figure 7c. We further emphasize that only those D_2_ molecules are observable in the deuterium 3p ESEEM experiments which are localized in the vicinity of the V^4+^ spin probes (≤1 nm) for a time of approximately at least 0.5 μs as determined by inverse deuterium Larmor frequency 1/*ν*_D_. The obtained parameters *k*_mod_ and *I*_mod_ exhibit a first lowering trend between 10–25 K indicating a decrease in the density of D_2_ molecules, which are trapped in the vicinity of the V^4+^ ions. More drastic changes are noticeable at 30 K when *k*_mod_ dropped down to approximately half of the *k*_mod_ value at 25 K. A similar trend can be deduced from the intensities *I*_mod_ in the 3p FT ESEEM spectra. We may conclude that at this temperature the majority of the adsorbed D_2_ molecules are no longer localized in the vicinity of the V^4+^ spin probes on the time scale (0.5 μs) of the deuterium ESEEM experiment and presumably diffuse rapidly through the pore system. Interestingly, the D_2_ TDS data (see section 2.3), indicate at 30 K the onset of the desorption process into the gas phase. Therefore, it seems justified to assume that the observed drop in *k*_mod_ and *I*_mod_ observed in the deuterium ESEEM experiments at 30 K is the microscopic manifestation of the beginning D_2_ desorption process.

The temperature‐dependent 3p ESEEM experiments reveal that the majority of D_2_ molecules are becoming already mobile on a microscopic scale within the pore system of the MOF framework at temperatures significantly lower in comparison to the macroscopically detectable desorption process by TDS. Similar behaviour has already been reported for physisorbed nitric oxide in various zeolites where the corresponding desorption energies could be determined from the temperature‐dependent EPR line width of the adsorbed NO molecules.^[36]^ Future work will aim at the development of theoretical approaches to extract similar information from the ESEEM spectra of adsorbed D_2_ gas.

In addition, we like to emphasize, that a minor part of the D_2_ molecules remains localized in the pores near the V^4+^ sites at temperatures up to 40 K as evidenced by weak deuterium modulation in the ESEEM spectra at these temperatures indicating a non‐vanishing D_2_ density there. Furthermore, the D_2_ density decreases already at *T*<30 K where a sudden drop in *k*_mod_ was observed. These results indicate a variety of physisorption sites for D_2_ in MIL‐53 (Al) having distinct desorption energies.

## Conclusions

In conclusion, we have demonstrated for the first time that X‐band *in situ* CW‐EPR spectroscopy can be implemented to directly (from the perspective of the framework) monitor the selective adsorption of dihydrogen isotopologues on flexible MOF materials such as MIL‐53 (Al_0.99_Cr_0.01_) and MIL‐53 (Al_0.99_V_0.01_). These experiments confirm the isotope‐selective phase transition for MIL‐53 (Al) via the 2^nd^ transition step in response to D_2_ adsorption at 23 K. Although the doping of Cr^3+^ and V^4+^ ions into the Al^3+^ site mainly induces a variation of the np to lp ratio, the two spin probes still offer comparable sensitivity to monitor this isotope‐selective phase transition. EPR parameters such as zero‐field splitting (for the Cr^3+^ probe), *g*‐tensors and hyperfine coupling (for the V^4+^ probe) serve as indicators of breathing transitions in the flexible MIL‐53 (Al) during dihydrogen isotope adsorption and desorption. Moreover, pulsed EPR techniques (3p ESEEM and HYSCORE) provide a more detailed picture of the host‐guest interaction between the V^4+^ spin probe embedded in the framework and the adsorbed D_2_ molecules. Temperature‐dependent 3p ESEEM spectroscopy provides insight into the local density of the adsorbed D_2_ species in the pores of the MIL‐53 (Al_0.99_V_0.01_) in the vicinity of the V^4+^ probes on the time scale of the deuterium ESEEM experiment. The drop in the deuterium modulation at about 30 K observed for the MIL‐53 (Al_0.99_V_0.01_) points to drastically enhanced mobility of the D_2_ molecules in the pore system, which is related to the onset of the desorption process and is coherent with TDS results even if the desorption is carried out under dynamic vacuum in the later experiment. In that way pulsed EPR spectroscopy, in particular ESEEM, allows to probe the D_2_ desorption for such porous materials on a local microscopic scale within the pore system as an alternative method to the established investigation methods in this research field that relies on gas phase detection.

The perspective on utilizing *in situ* pulsed EPR setup to detect the dihydrogen isotopologues (H_2_ and D_2_) in a 1 : 1 mixture is currently in progress. Apart from the knowledge about the shifts in breathing transition pressure due to different D_2_ partial pressure in the H_2_/D_2_ mixtures than in the pure D_2_ case (if the *in situ* CW EPR is conducted), it is expected that the higher resolution of ESEEM spectroscopy would be useful to study the host‐guest interaction during the adsorption and desorption of D_2_ molecules from the gas mixtures based on the changes on weak dipolar interactions of distant deuterium nuclei when compared to the adsorption of pure dihydrogen gas. However, it is also important to address the issue that the para‐H_2_ species with total nuclear spin *I*=0 that occurs at cryogenic temperatures would escape the ESEEM detection.

## Experimental Section

### Sample Preparation

MIL‐53 (Al_0.99_Cr_0.01_) was prepared by hydrothermal synthesis according to the procedure from Mendt and co‐workers.^[18]^ The synthesis protocol was conducted using a 27 mL Teflon‐lined steel autoclave. AlCl_3_ ⋅ 6H_2_O (1.30 g, 5.4 mmol), Cr(NO_3_)_3_ ⋅ 9H_2_O (0.0139 g, 0.034 mmol), and terephthalic acid (0.5 g, 3.0 mmol) were mixed and dissolved in 5 mL of water. The hydrothermal synthesis was implemented at 423 K for 24 h. The crystals were filtered off after the reaction finished and dried under an ambient atmosphere.

The synthesis of MIL‐53 (Al_0.99_V_0.01_) MOF was done using the hydrothermal synthesis method described in the work of Nevjestic and coworkers,[^21^, ^37^] where the synthesis was performed by mixing AlCl_3_ ⋅ 6H_2_O (0.74 g, 5.55 mmol), VCl_3_ (0.0089 g, 0.057 mmol), and terephthalic acid (0.232 g, 3.0 mmol) in a 27 mL Teflon‐lined steel autoclave. The reaction was deployed for 72 h and up to the temperature of 475 K. The resulting powder was placed into the autoclave with methanol to remove all unreacted reagents and dried for 24 h. The crystals were filtered off after the reaction finished and dried under an ambient atmosphere.

The *ex‐situ* D_2_ adsorption on MIL‐53 was conducted using a standard vacuum line which is connected to the sample tube. The sample was activated first at 475 K for 12 h under vacuum (ca. 10^−2^ mbar) and then exposed for 10 minutes to 133 mbar of D_2_ gas at 77 K. The sample tube was sealed while in contact with liquid nitrogen to avoid direct D_2_ desorption from the sample.

### *In Situ* CW‐EPR Experiments

The *in situ* CW‐EPR experiments were performed with a Bruker EMX micro (X‐band, 9.4 GHz) spectrometer equipped with an ER 4119 HS cylindrical cavity. For low‐temperature experiments, an Oxford Instruments Helium cryostat ESR 900 was used. Spectral simulations of the EPR spectra were performed using the MATLAB simulation package EasySpin.^[38]^ Both H_2_ and D_2_ Minican® gas with 99.999 % purity were purchased from Linde AG. A home‐built gas loading line was previously introduced by Mendt and co‐workers.^[39]^ This gas manifold setup allows the exposure of either H_2_ or D_2_ gas to the sample at a well‐defined pressure (pressure range 0.001 mbar–1000 mbar) and temperature, while the sample is kept in the cryostat of the EPR spectrometer (See Figure S11 for the schematic illustration of the setup). In the present work, the H_2_ or D_2_ pressure was increased stepwise, starting at a pressure of *p*=0.001 mbar until the saturation vapour pressure *p*_o_ was reached at the target temperature. Then, the pressure was gradually decreased down to *p*=0.001 mbar again for the desorption stage. In most cases, at each pressure, EPR spectra were measured continuously until further spectral changes were observed and the last one was considered to represent the desired equilibrium state.

### Pulsed EPR Experiments

Pulsed EPR experiments were conducted using a Bruker ELEXYS E580 spectrometer. The two‐pulse field‐swept electron spin‐echo (FSESE) spectra were recorded using a π/2‐*τ* ‐π‐*τ* ‐echo pulse sequence with a pulse delay of *τ=*200 ns. For three‐pulse electron spin echo envelope modulation (3p ESEEM), the pulse sequence π/2‐*τ* ‐π/2‐*T*‐π/2‐*τ* ‐echo was implemented with a pulse delay *τ* of 134 and 218 ns. The pulse delay between the second and the third π/2 pulses (*T*) was incremented by steps of 16 ns starting from 120 ns. The fast Fourier transformed (FFT) 3p ESEEM spectra were obtained via 3^rd^ order polynomial of baseline correction, hamming window apodization, zero‐filling to 1024 points and FT and presented as the absolute value FFT spectra. In addition, the pulse sequence of π/2‐*τ*‐π/2‐*t*_1_‐π‐*t*_2_‐π/2‐*τ*‐echo was employed for HYSCORE experiments. π/2 and π pulse lengths are 16 and 32 ns, respectively whereas the pulse delays *τ* are set to 134 and 218 ns. A 170x170 data matrix was sampled. The data from the HYSCORE experiments were post‐processed through 3rd order polynomial of baseline correction, hamming window apodization, zero‐filling to 1024 points, 2‐dimensional Fourier transformation and the absolute value extraction. The HYSCORE spectra were further treated and presented with a symmetrization function using the Hyscorean.^[40]^

### Thermal Desorption Spectroscopy Experiments

Thermal Desorption Spectroscopy (TDS) measurements were performed using a custom laboratory setup. This technique provides information on the binding energies of molecular species adsorbed on a solid surface. Typically, 3–4 mg of the sample is sufficient for these measurements. MIL‐53 (Al_0.99_Cr_0.01_) and MIL‐53 (Al_0.99_V_0.01_) samples were activated at 403 K for 12 hours before the measurement. Following activation, the sample is then cooled to 20 K and exposed to an atmosphere of H_2_ or D_2_ with varying exposure pressure (*P*_exp_) for 10 minutes. Subsequently, the sample is evacuated to remove any excess non‐adsorbed gas. A linear heating ramp of 0.1 K/s is then applied. As the sample is heated from 20 K to 200 K, the desorbing gas is continuously monitored using a quadrupole mass spectrometer. To quantify the mass spectrometer signal, the TDS device was calibrated with a Pd_95_Ce_5_ alloy^[41]^ loaded with hydrogen or deuterium, quantifying that the area under the curve is proportional to the amount of gas desorbed.

## Conflict of Interests

The authors declare no conflict of interest.

1

## Supporting information

As a service to our authors and readers, this journal provides supporting information supplied by the authors. Such materials are peer reviewed and may be re‐organized for online delivery, but are not copy‐edited or typeset. Technical support issues arising from supporting information (other than missing files) should be addressed to the authors.

Supporting Information

## Data Availability

The data that support the findings of this study are openly available in Zenodo at https://doi.org/10.5281/zenodo.13837026, reference number 13837026.
