# Supplementary material for: In Situ Electron Paramagnetic Resonance Investigation of Isotope‐Selective Breathing in MIL‐53 During Dihydrogen Adsorption
Source: Chemistry. 2025 Jan 31;31(13):e202500088. doi: 10.1002/chem.202500088 (PMC11874685; doi:10.1002/chem.202500088)
Supplement: Supplementary file 1 — Supporting Information [file CHEM-31-e202500088-s001.pdf]

Chemistry–A European Journal

Supporting Information

In Situ Electron Paramagnetic Resonance Investigation of Isotope-Selective Breathing in MIL-53 During Dihydrogen Adsorption

Muhammad Fernadi Lukman, Sibo Chetry, Prantik Sarkar, Volodymyr Bon, Kavipriya Thangavel, Stefan Kaskel, Michael Hirscher, Harald Krautscheid, and Andreas Pöppl*

Supplementary Information

In situ Electron Paramagnetic Resonance Investigation of Isotope-Selective Breathing in MIL-53 During Dihydrogen Adsorption

Muhammad Fernadi Lukman[a], Sibo Chetry[b], Prantik Sarkar[c,f], Volodymyr Bon[d], Kavipriya Thangavel[e], Stefan Kaskel[d], Michael Hirscher[c,g], Harald Krautscheid[b] and Andreas Pöppl[a*]

[a] Felix Bloch Institute for Solid State Physics, Leipzig University, Leipzig 04103, Germany
[b] Faculty of Chemistry and Mineralogy, Institut for Inorganic Chemistry, Leipzig University, Leipzig 04103, Germany
[c] Max Planck Institute for Intelligent Systems, Stuttgart 70569, Germany
[d] Chair of Inorganic Chemistry I, Technical University Dresden, Dresden D-01069, Germany
[e] National High Magnetic Field Laboratory, Florida State University, Tallahassee 32310, USA
[f] Institute of Separation Science and Technology, Friedrich-Alexander-Universität Erlangen-Nürnberg (FAU), Erlangen 91058, Germany
[g] Advanced Institute for Materials Research (WPI-AIMR), Tohoku University, Aoba-ku, Sendai, 980-8577, Japan
*Corresponding Authors, E-mail: poeppl@physik.uni-leipzig.de

1.1 *In situ* variable temperature-PXRD experiments

In situ variable temperature PXRD patterns in vacuum ($p < 10^{-4}$ mbar) were measured on Empyrean-2 powder X-ray diffractometer (ω–2θ goniometer, alpha1 system) using a customized setup based on ARS DE-102 closed-cycle helium cryostat (T = 30 – 300 ± 0.1 K) and adsorption cell, built of 1.33″ CF-flange and Beryllium dome. The cell was connected to the low-pressure port of the BELSORP-max (Microtrac MRB) volumetric adsorption instrument to ensure the ultra-high vacuum in the measurement cell. The diffraction experiments were conducted using ω–2θ scans in transmission geometry in the range of 2θ = 2–70°. After reaching the target temperature the sample was equilibrated for 15 minutes. Parallel beam optics (W/Si mirror, hybrid 2xGe(220) monochromator, 4 mm mask, primary divergence and secondary anti-scatter slits with 0.5° opening) was used for the data collection. A PixCEL-3D detector in 1D scanning mode (255 active channels) was used to record the scattered intensities. PXRD patterns are plotted in Figure S1. The reflections with high intensities in the range of 2θ = 44 – 55° and 2θ = 68° originated from the beryllium dome. The quantitative phase analysis was conducted using Reflex Tool of Materials Studio software assuming the presence of exclusively crystalline phases in the sample. The crystal structures of MIL-53(Al)-lp and MIL-53(Al)-np were used for the data analysis.

Variable temperature PXRD analysis without presence of H_2 or D_2 gas has been conducted to confirm the phase purity of doped MIL-53 ($Al_{0.99}Cr_{0.01}$) and MIL-53 ($Al_{0.99}V_{0.01}$) samples. PXRD patterns for both samples were collected in a vacuum upon cooling down from 300 K to 30 K and warming up again with 20 K steps (Figure S1). The phase analysis revealed the phase transition from lp to np in the temperature range of 170 - 70 K indicating an increase in the weight percentage of the np phase from 3.5 to 89.7 wt.% (Figure S2a). The heating of the sample from 30 to 300 K imparts almost negligible changes in the phase composition, reflecting the stabilization of the

np metastable phase. At 300 K the ratio of lp to np phases evolves from 85.8 to 14.2 wt.%. Such large temperature-driven hysteresis and reversibility are in line with previously reported temperature-induced breathing in MIL-53 (Al).[1,2] A careful analysis of the unit cell volume of the phases shows a slight reduction of the unit cell volume for both lp and np phases as the measurement temperature decreases (See Figure S2 b,c).

A distinctive behaviour was observed in the case of MIL-53 ($Al_{0.99}V_{0.01}$), in which the lp to np phase transition is shifted towards a lower temperature. Specifically, the percentage of the np phase only increases from 3.9 to 22.5 wt.% upon cooling the sample from 90 to 30 K (Figure S2d). Upon heating, the percentage of the np phase stays nearly unchanged and start to decrease at 300 K to 21.3%. The evolution of unit cell volume for lp and np phase are given in Figure S2e,f indicating the unit cell volume slightly decreases for the np phase during cooling to 100 K. Overall, these preliminary variable temperature PXRD data demonstrate that MIL-53 ($Al_{0.99}V_{0.01}$) tends to exhibit more co-existence of np and lp phases if compared to MIL-53 ($Al_{0.99}Cr_{0.01}$) at the same temperature.

1.2 *In situ* CW-EPR spectral analysis

The characteristic X-band CW-EPR spectra of Cr^{3+} ions (electronic configuration: $3d^3$) at various adsorbate pressures have been simulated using Easyspin software[3] considering an electron spin S = 3/2 using a spin-Hamiltonian operator

$$\hat{H} = \beta_e \vec{B} \boldsymbol{g} \hat{\vec{S}} + \hat{\vec{S}} \boldsymbol{D} \hat{\vec{S}} \qquad (1)$$

where the first term describes the Zeeman interaction between the electron spin S = 3/2 of Cr^{3+} and the external magnetic field as a result of matrices multiplications between the external magnetic field vector $\vec{B}$, the ***g***-tensor, and the electron spin vector operator $\hat{\vec{S}}$. In addition, the constant β_e is the Bohr magneton. The second term yields the zero field splitting (ZFS) of the Cr^{3+} ion with the ZFS tensor ***D***. The principal values D_{ii} of the traceless tensor ***D*** can be represented by only two parameters, the axial ($D = 3D_{zz}/2$) and orthorhombic ($E = (D_{xx} - D_{yy})/2$) ZFS parameters.[2,4] From the spectral simulation, we assumed the ***g***-tensor is isotropic with only minor changes of its value for the different phases of MIL-53 ($Al_{0.99}$ $Cr_{0.01}$)[2,4] whereas the hyperfine interaction with the ^{53}Cr isotope (nuclear spin I = 3/2 and natural abundance of 9.55%) was neglected. The principal values of zero-field splitting are very sensitive for exploring phase transformations as a response to gas adsorption in the MIL-53 framework.[4]

All of the V^{4+} *in-situ* CW-EPR spectra related to V^{4+} are also simulated using the Easyspin toolbox[3] with the spin Hamiltonian operator

$$\hat{H} = \beta_e \vec{B} \boldsymbol{g} \hat{\vec{S}} + \hat{\vec{S}} \boldsymbol{A}^{\boldsymbol{V}} \hat{\vec{I}}^{V} \qquad (2)$$

The first term in eq. (2) represents again the Zeeman interactions of the V^{4+} electron spin S = ½. The second term is related to the hyperfine (HF) interaction between the electron spin and the ^{51}V nuclei having a nuclear spin I^V = 7/2, where $\hat{I}^V$ is the nuclear

spin operator and $\boldsymbol{A^V}$ the vanadium HF coupling tensor with principal values A_i^V $(i = x, y, z)$.

A quantitative approach to calculate the relative amount of the molar fraction of three pore phases (np, lp and vlp) can be implemented by simulating the experimental CW-EPR spectra. We introduce the weighing factors I_{np}, I_{lp} and I_{vlp} for the simulation of the total spectrum in the following equation

$$S_{sum} = I_{np}S_{np} + I_{lp}S_{lp} + I_{vlp}S_{vlp} \ , \qquad (3)$$

where S_{np}, S_{lp} and S_{vlp} are the simulated spectra of the np, lp and vlp pore phases calculated with Easyspin software.[3] The estimated error for the molar fraction is determined to be 4% in the region of p = 0.001 mbar to 10 mbar and < 4% when measured at p > 10 mbar, respectively.

1.3 Pulsed EPR spectral analysis

The simulation of HYSCORE spectra was conducted using Easyspin[3] by implementing the following spin Hamiltonian:

$$\hat{H} = \beta_e \vec{B} \boldsymbol{g} \hat{\vec{S}} + \hat{\vec{S}} \boldsymbol{A^V} \hat{\vec{I}}^V + \sum_i \hat{\vec{S}} \boldsymbol{A^i} \overrightarrow{\hat{I}^i} - \sum_i \beta_n \boldsymbol{g_n^i} \vec{B} \hat{\vec{I}}^i + \sum_i \hat{\vec{I}}^i \boldsymbol{Q}^i \hat{\vec{I}}^i \qquad (4)$$

The first term describes the electron Zeeman interaction of the electron spin S = ½ with its spin vector operator $\hat{\vec{S}}$ and $\boldsymbol{g}$-tensor of the V^{4+} ion and the external magnetic field $\vec{B}$. The second term implements the hyperfine (hf) interaction between the interaction tensor $\boldsymbol{A^V}$ of the electron spin with the ^{51}V nuclear spin I_V = 7/2 where $\hat{\vec{I}}^V$ is the corresponding nuclear spin vector operator. The fourth term takes into account the ligand HF couplings and nuclear Zeeman interactions of the nuclear spins I_i of ^{27}Al (I_{Al} = 5/2), ^2D (I_D = 1) and ^1H (I_H = 1/2) in the second or higher coordination spheres surrounding the V^{4+} ion. $\boldsymbol{A^i}$ and g_n^i are the corresponding shf interaction tensors and nuclear g factors, respectively. The hyperfine matrix $\boldsymbol{A^i}$ consists of an isotropic component, $a_{iso}^i$and an anisotropic component $\boldsymbol{T^i}$ with principal values $T_{xx}^i, T_{yy}^i, T_{zz}^i$ in the following equation: $\boldsymbol{A^i} = a_{iso}{}^i \mathbf{1} + \boldsymbol{T^i}$ where $\mathbf{1}$ is the identity matrix. In the case of the ^{27}Al simulation, the last term comprises of ^{27}Al nuclear quadrupole interaction of the ligand nuclei with the NQ coupling tensors $\boldsymbol{Q}^i$. The principal values for the traceless nq tensor are usually parametrized by the NQ coupling constant e^2qQ/h and the asymmetry parameter η (where 0 < η < 1).

HYSCORE[5] spectra were recorded before and after the *ex-situ* adsorption of D_2 gas on the MIL-53 ($Al_{0.99}$ $V_{0.01}$) sample. Figure S7a illustrates the spectrum of activated MIL-53 ($Al_{0.99}$ $V_{0.01}$) before D_2 adsorption and it demonstrates the presence of ^1H cross peaks extending from (12.07,18.16) MHz to (18.16,12.07) MHz and centered at the ν_H = 14.74 MHz which can be ascribed to the μ_2-OH next-neighboured to the V^{4+} ion.[6] In addition, a small broad peak at 3.85 MHz on the diagonal is assigned to aluminium (Al^{3+}) from next-neighboured octahedral framework sites.

The simulation of this proton and the aluminium signals at the activated state of the MIL-53($Al_{0.99}$ $V_{0.01}$) sample are given in Figure S7-10 while the spin Hamiltonian parameters of weakly-coupled nuclei are tabulated in Table S1. Our simulated ^{1}H and ^{27}Al HF coupling parameters are in good agreement with those reported by Nevjestic and coworkers using Q-band electron-nuclear double resonance (ENDOR) spectroscopy.[6] On the other hand, the simulation of the ^{27}Al cross peak allows an estimation of the upper limit of the aluminium nuclear quadrupolar (NQ) coupling constant e^2qQ/h of 6 MHz and an asymmetry parameter η = 0.1 which is consistent with the previously reported NQ parameters for the ^{27}Al site in the pure MIL-53(Al) (e^2qQ/h = 7 MHz, η = 0.1) probed by magic angle spinning (MAS) NMR.[7]

Please note that D_2 molecules in the gas phase may exhibit different quantum rotational states: ortho-D_2 (even rotational states) and para-D_2 (odd rotational states). Considering our current measurement at 10 K, J = 0 rotational ground state (ortho-D_2) having a nuclear spin configuration I = 2 is the most populated state.[8] Hence, we may assume that D_2 molecules most likely possess an I = 2 nuclear spin configuration which would allow the detection of a ^{2}D modulation of the adsorbed D_2 molecules with weak HF couplings in the 3p ESEEM and HYSCORE experiments after the D_2 adsorption on MIL-53($Al_{0.99}$ $V_{0.01}$). On the contrary, we can predict that adsorbed H_2 molecules will have a predominantly para-H_2 state with a total nuclear spin equal to zero,[9,10] thus it is not possible to detect a ^{1}H modulation of adsorbed H_2 molecules with small HF couplings near the V^{4+} spin probes at our measurement conditions. Therefore, 3p ESEEM and HYSCORE investigations were only performed in the case of D_2 adsorption over MIL-53($Al_{0.99}$ $V_{0.01}$). The HYSCORE spectrum after adsorption of D_2 was recorded with τ = 218 ns for ^{2}D signal enhancement. The spectra exhibit an intensive cross peak at the deuterium nuclear Larmor frequency ν_D = 2.21 MHz indicating the presence of D_2 in the lp phase. HYSCORE simulations of the ^{2}D signal (Figure S10) yield only small deuterium HF coupling parameters and a negligible ^{2}D NQ interaction (Table S1). The latter is expected for the spherical symmetric J = 0 ground state of D_2. The estimated small dipolar ^{2}D HF coupling parameter T = 0.1 MHz indicates that the D_2 molecules are not directly coordinating with the distorted vanadium octahedra but are located at a distance of approximately 0.49 nm or further from our V^{4+} spin probes. Moreover, we would also like to point out a very high intensity of the HYSCORE signal of the adsorbed D_2 molecules if compared to the ^{1}H and ^{27}Al signals of the framework protons. Although not in a quantitative manner, it essentially proves the adsorption of a significant number of D_2 molecules in the pores. However, we should also consider cross-suppression effect from ^{2}D might also contribute to the weakening of the ^{1}H signal.[11]

Table S1. Simulated spin Hamiltonian parameter of weakly-coupled nuclei identified from HYSCORE spectral analysis of the activated MIL-53 ($Al_{0.99}V_{0.01}$) sample and the *ex situ* D_2 adsorbed on MIL-53 ($Al_{0.99}V_{0.01}$). α, β, ɣ are the Euler angles of the ***A*** tensor with respect to the ***g*** tensor.

Nuclei	a_{iso} / MHz	T / MHz	α, β, γ / degree	e^2qQ/h / MHz	η
**^{1}H** (-O**H** bridge)	0.5	3.5	0,55,0	-	-

**^{27}Al**	0.1-0.4	1.1	0,0,0	6	0.1
**^{2}D**	0.01-0.1	0.1	0,0,0	-	-

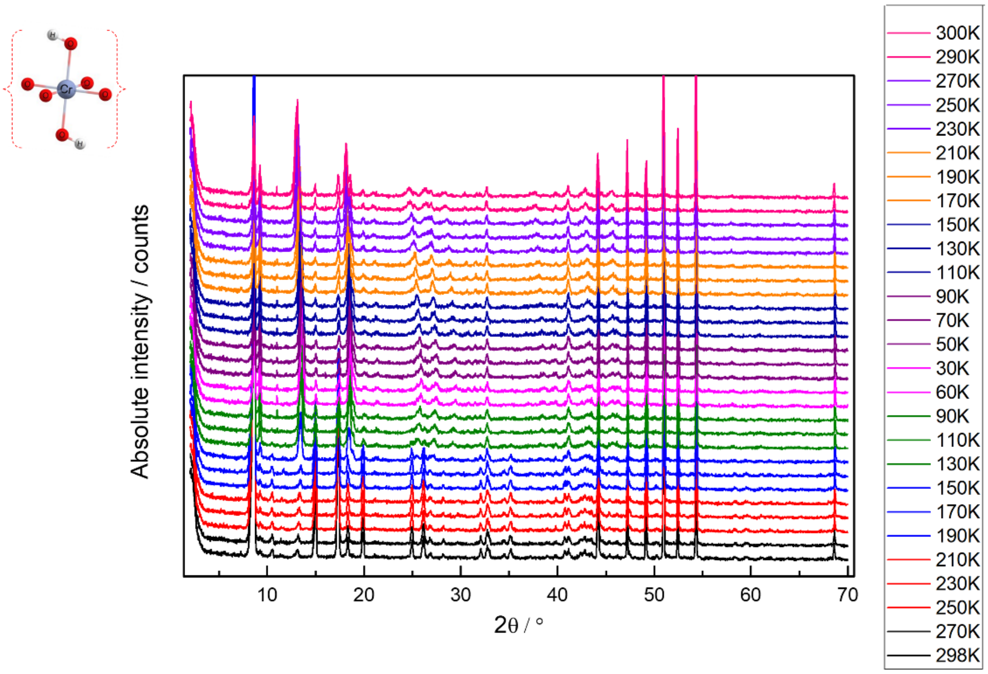

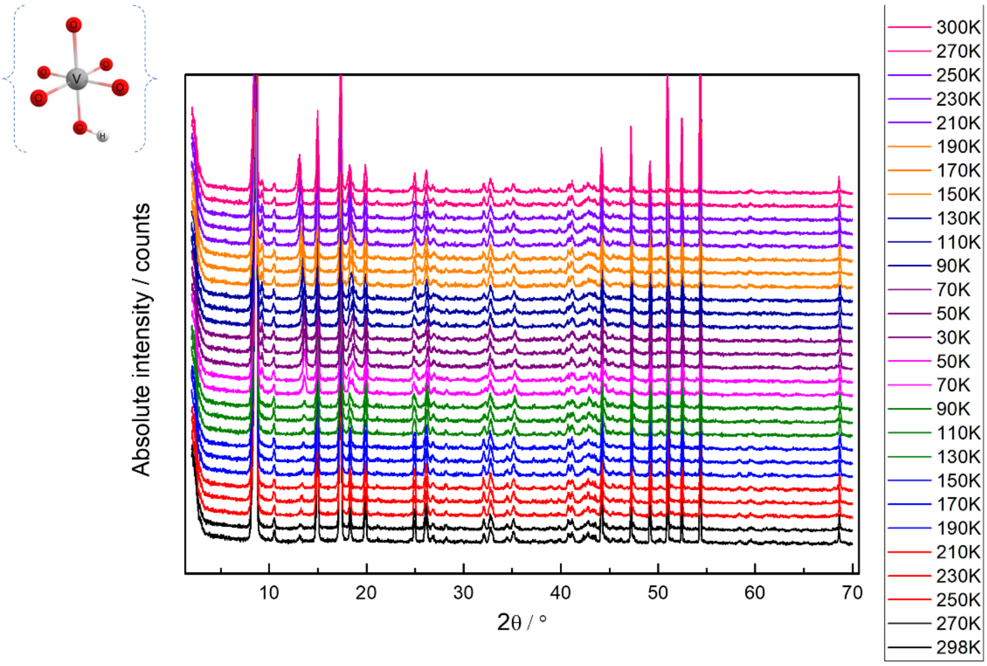

Figure S1. Variation temperature of PXRD patterns for MIL-53 ($Al_{0.99}$ $Cr_{0.01}$) (top) and MIL-53 ($Al_{0.99}$ $V_{0.01}$) (bottom) samples obtained under vacuum conditions.

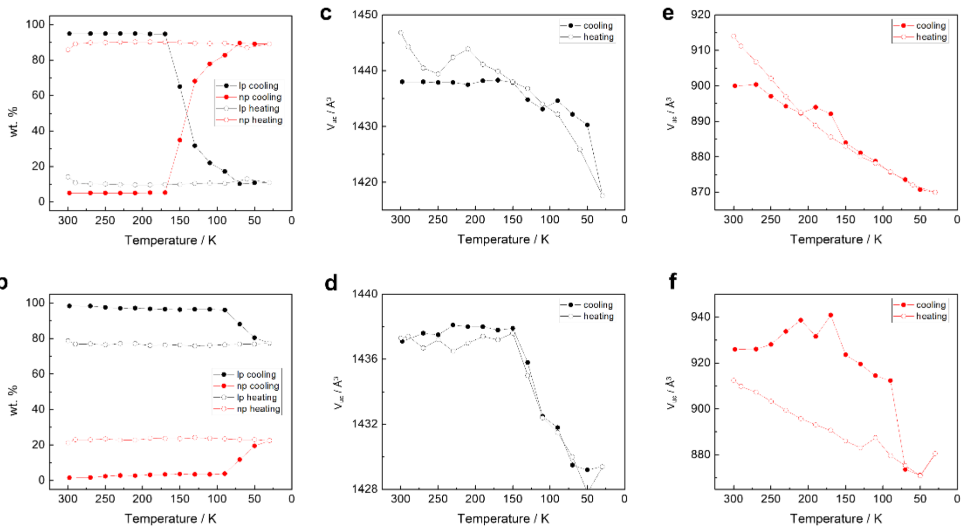

Figure S2. Quantitative phase analysis of (a) MIL-53 ($Al_{0.99}Cr_{0.01}$), (b) MIL-53 ($Al_{0.99}V_{0.01}$) and temperature dependence of unit cell volume, V_{uc} for the lp phase in (c) MIL-53 ($Al_{0.99}Cr_{0.01}$) and (d) MIL-53($Al_{0.99}V_{0.01}$). In addition, the temperature dependence of V_{uc} for the np phase in (e) MIL-53 ($Al_{0.99}Cr_{0.01}$) and (f) MIL-53($Al_{0.99}V_{0.01}$) is also presented.

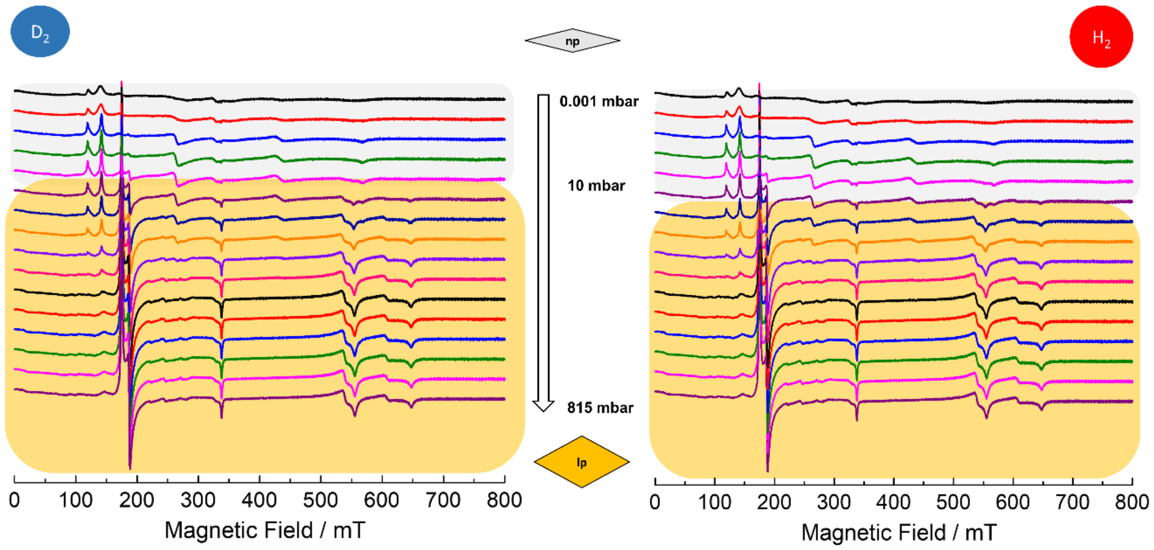

Figure S3. *In situ* CW-EPR spectra spectra recorded during D_2 (left panel) and H_2 gas (right panel) adsorption on MIL-53 ($Al_{0.99}$ $Cr_{0.01}$) at 40 K.

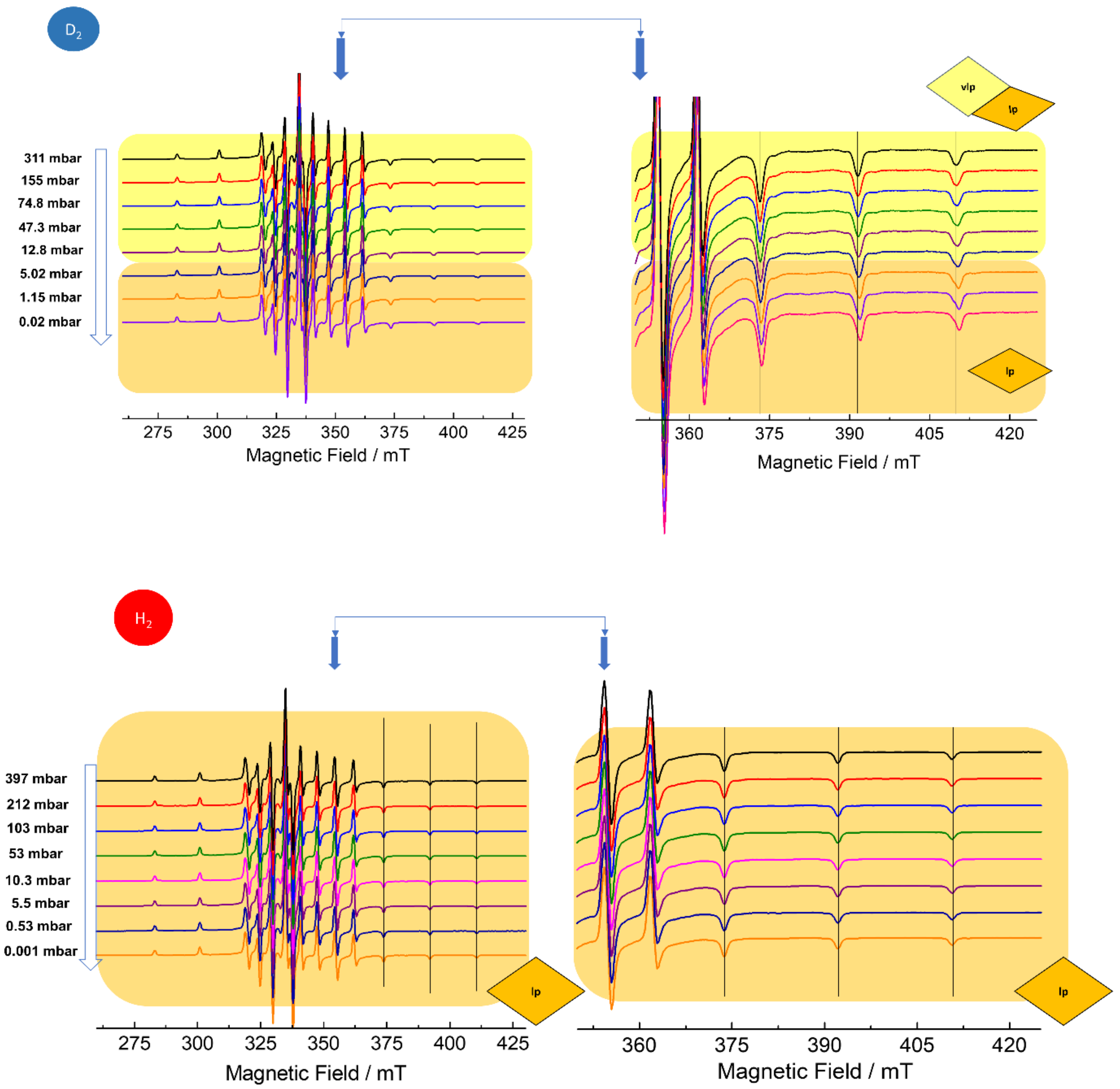

Figure S4. *In situ* CW-EPR spectra of MIL-53($Al_{0.99}$ $V_{0.01}$) during desorption stages of D_2 at 23 K (top) and H_2 gas at 20 K (down).

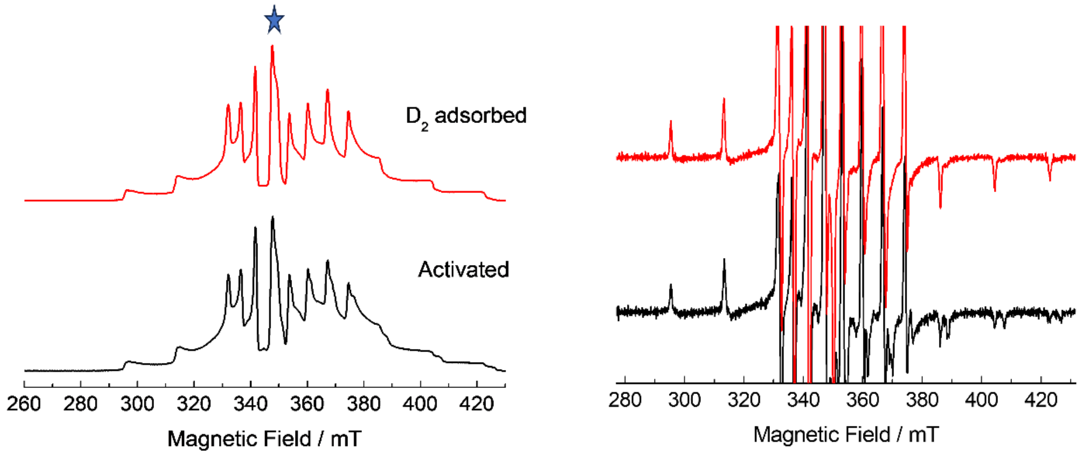

Figure S5. Echo-detected field sweep (EDFS) spectra (a) for the activated MIL-53 ($Al_{0.99}V_{0.01}$) and D_2 adsorbed MIL-53 ($Al_{0.99}V_{0.01}$) alongside their corresponding first derivative spectra (b). The star marks the field position for the following experimental HYSCORE spectra.

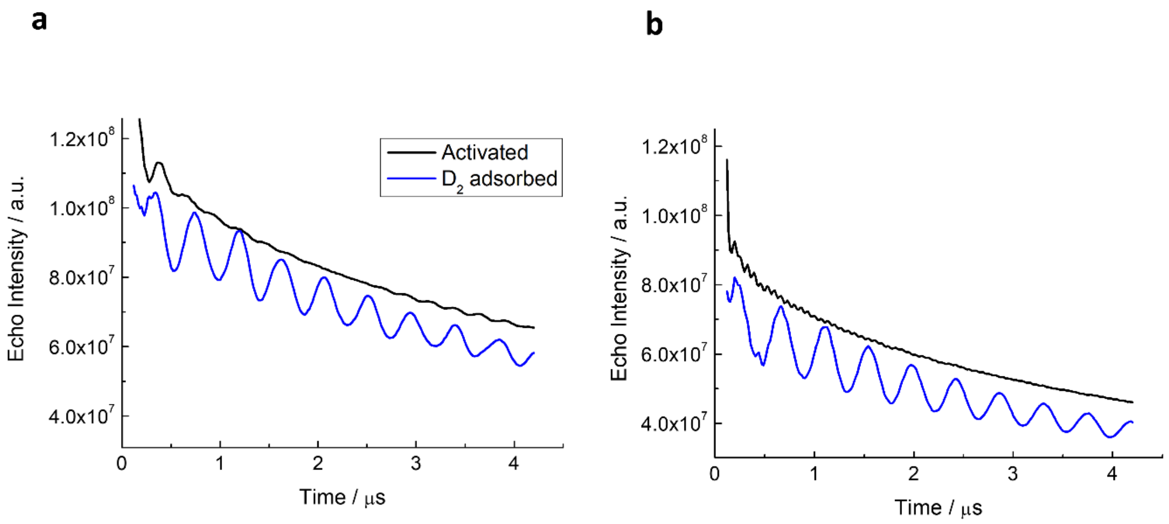

Figure S6. Three-pulse (3p) ESEEM spectra for the activated MIL-53 ($Al_{0.99}V_{0.01}$) (in black) and the *ex-situ* D_2 adsorbed MIL-53 ($Al_{0.99}V_{0.01}$) (in blue) recorded at a pulse delay of 134 ns (a) and 218 ns (b). All spectra were measured at 10 K.

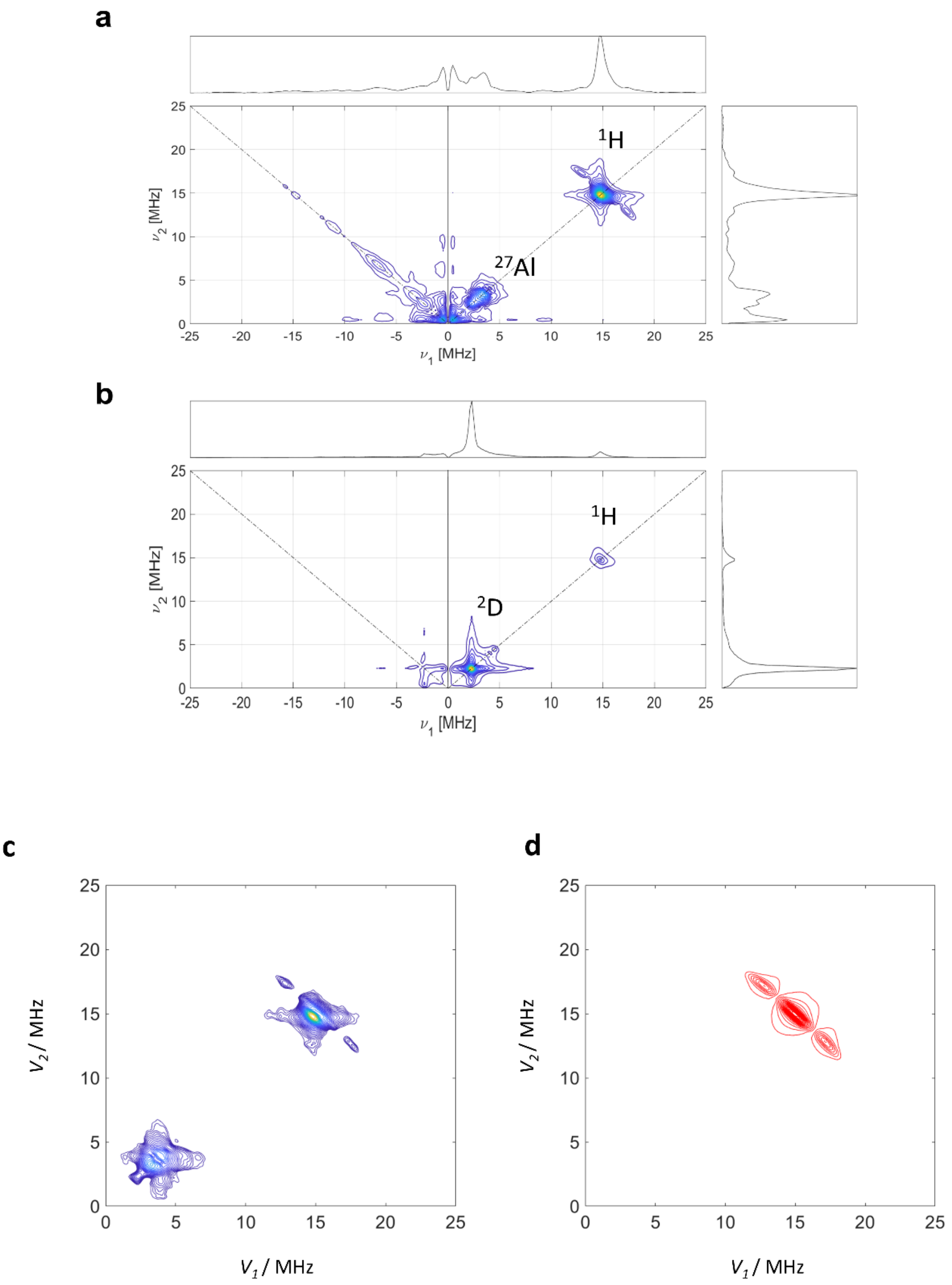

Figure S7. HYSCORE spectra of the activated MIL-53($Al_{0.99}V_{0.01}$) sample (a), HYSCORE spectra of D_2 adsorbed MIL-53($Al_{0.99}V_{0.01}$) sample (b). Both were recorded at 10 K at a pulse delay of 218 ns and magnetic field position of 347 mT. HYSCORE spectra using a pulse delay of 218 ns at 10 K (c) for the activated MIL-53 ($Al_{0.99}$ $V_{0.01}$) and its spectral simulation for the proton signal (d).

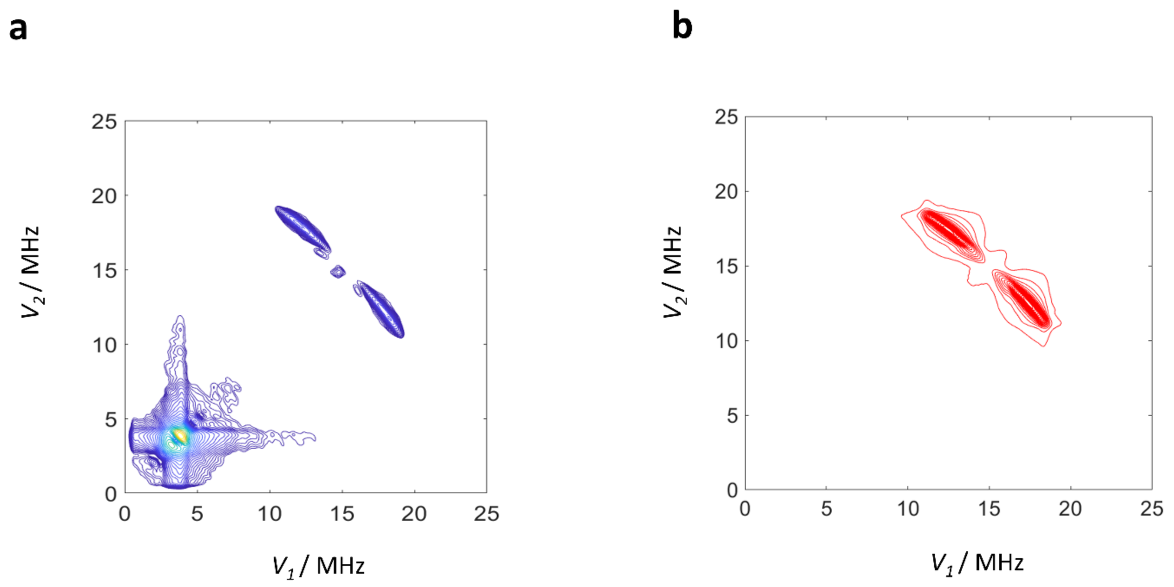

Figure S8. HYSCORE spectra using a pulse delay of 134 ns at 10 K (a) for the activated MIL-53 ($Al_{0.99}V_{0.01}$) and its spectral simulation for the proton signal (b).

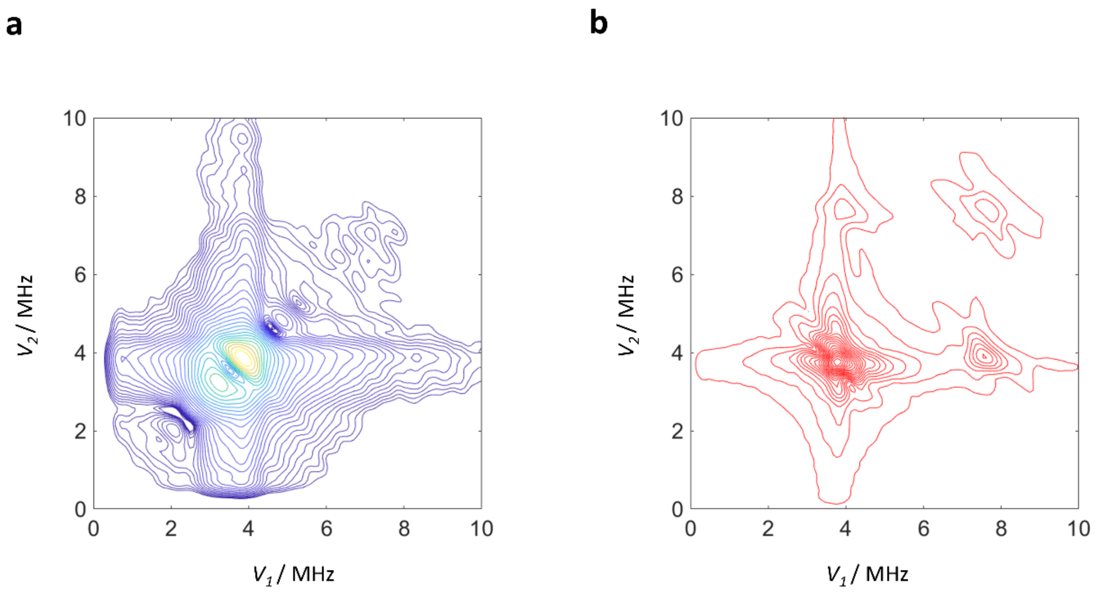

Figure S9. HYSCORE spectra using a pulse delay of 134 ns at 10 K (a) for the activated MIL-53 ($Al_{0.99}V_{0.01}$) and its spectral simulation (b) for the aluminium signal.

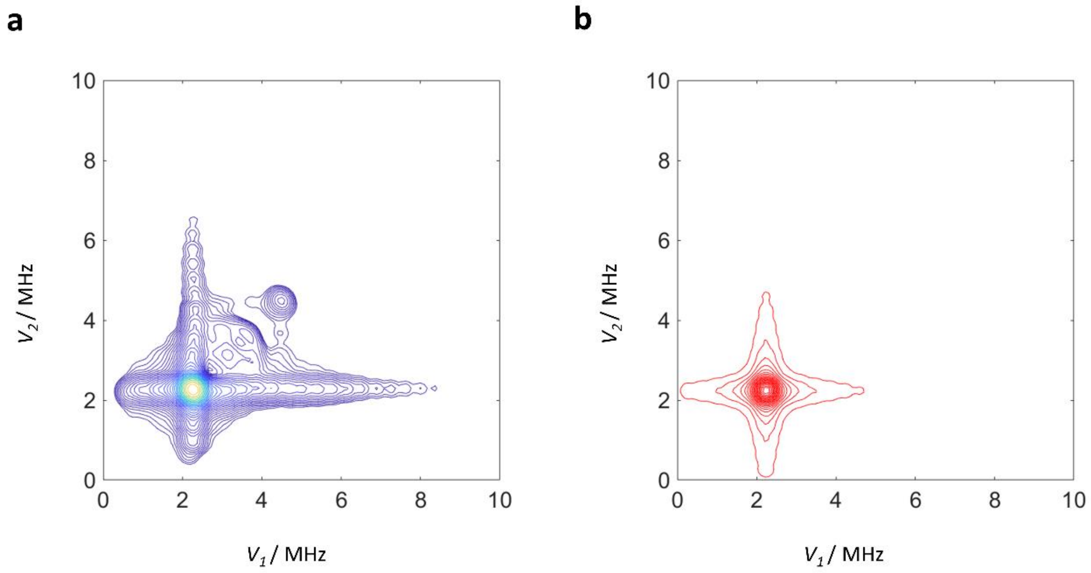

Figure S10. HYSCORE spectra using a pulse delay of 218 ns at 10 K (a) for the D_2 adsorbed MIL-53 ($Al_{0.99}V_{0.01}$) and its spectral simulation (b) for the distant deuterium signal.

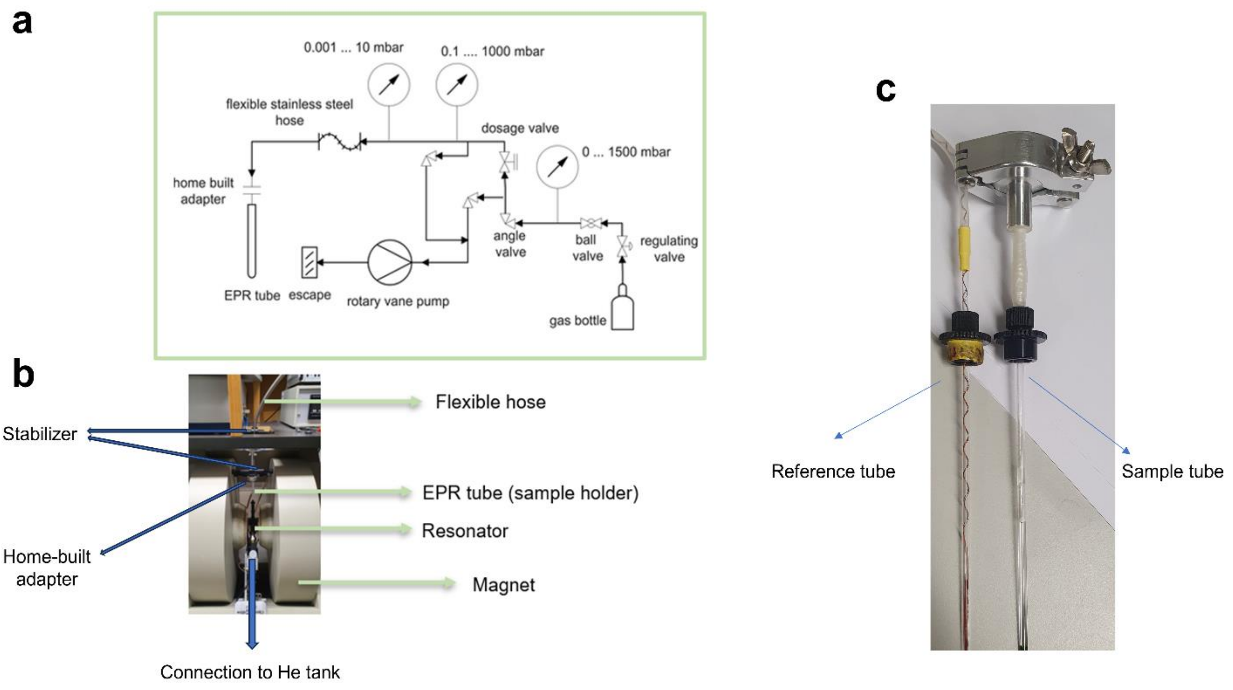

Figure S11. a) The schematic workflow of gas loading setup[12] for the in situ EPR measurement, b) A close view of the gas manifold connection to the resonator of CW-EPR spectrometer and c) a photograph of the sample quartz tube connection with the home-built adapter as well as the reference tube for an accurate temperature check.

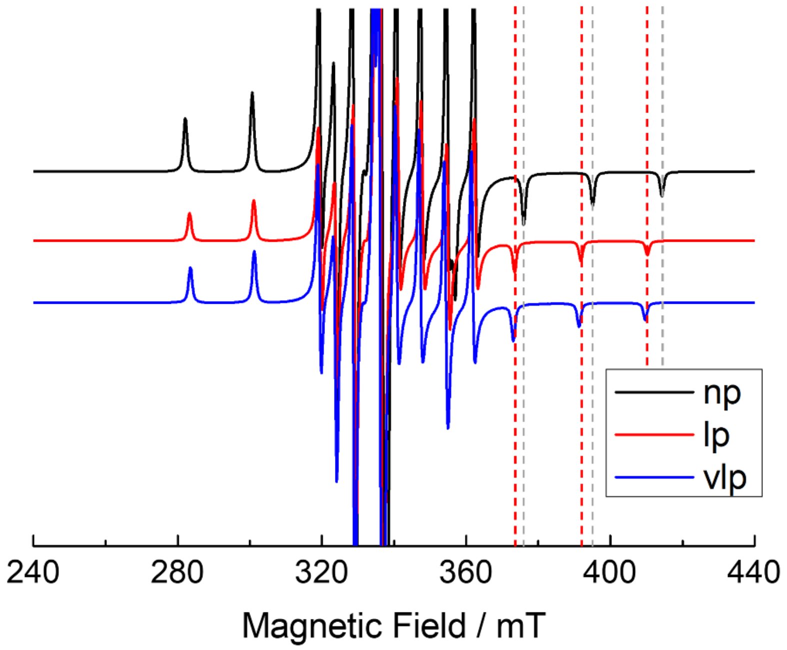

Figure S12. A comparison of simulated EPR spectra in one axis for species np, lp and vlp.

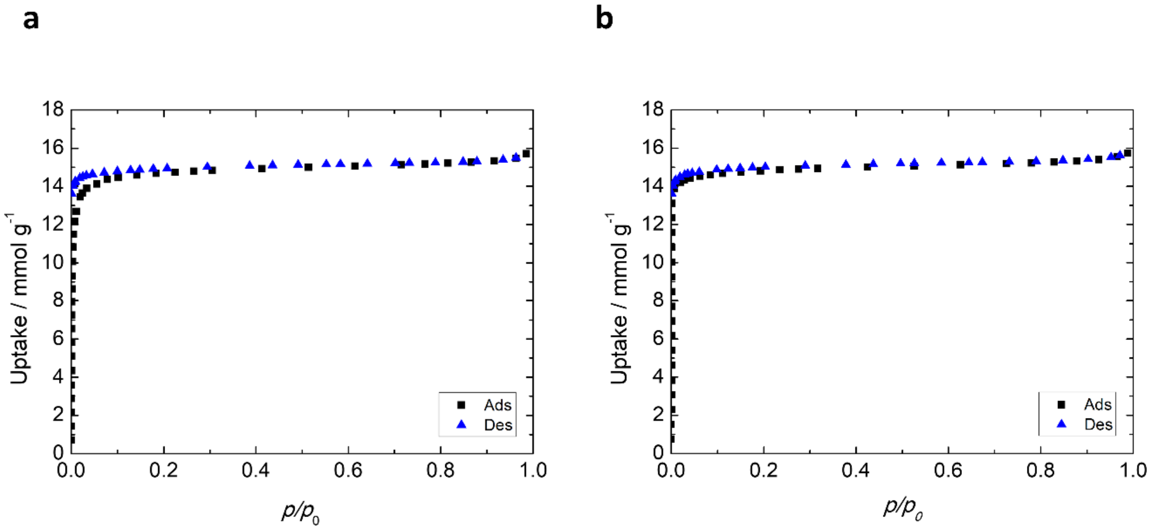

Figure S13. N_2 sorption isotherm for a) MIL-53 ($Al_{0.99}Cr_{0.01}$) and b) MIL-53 ($Al_{0.99}V_{0.01}$) samples measured at 77 K. The BET surface are 1559 and 1360 m^2 g^{-1} for MIL-53 ($Al_{0.99}Cr_{0.01}$) and MIL-53 ($Al_{0.99}V_{0.01}$), respectively.

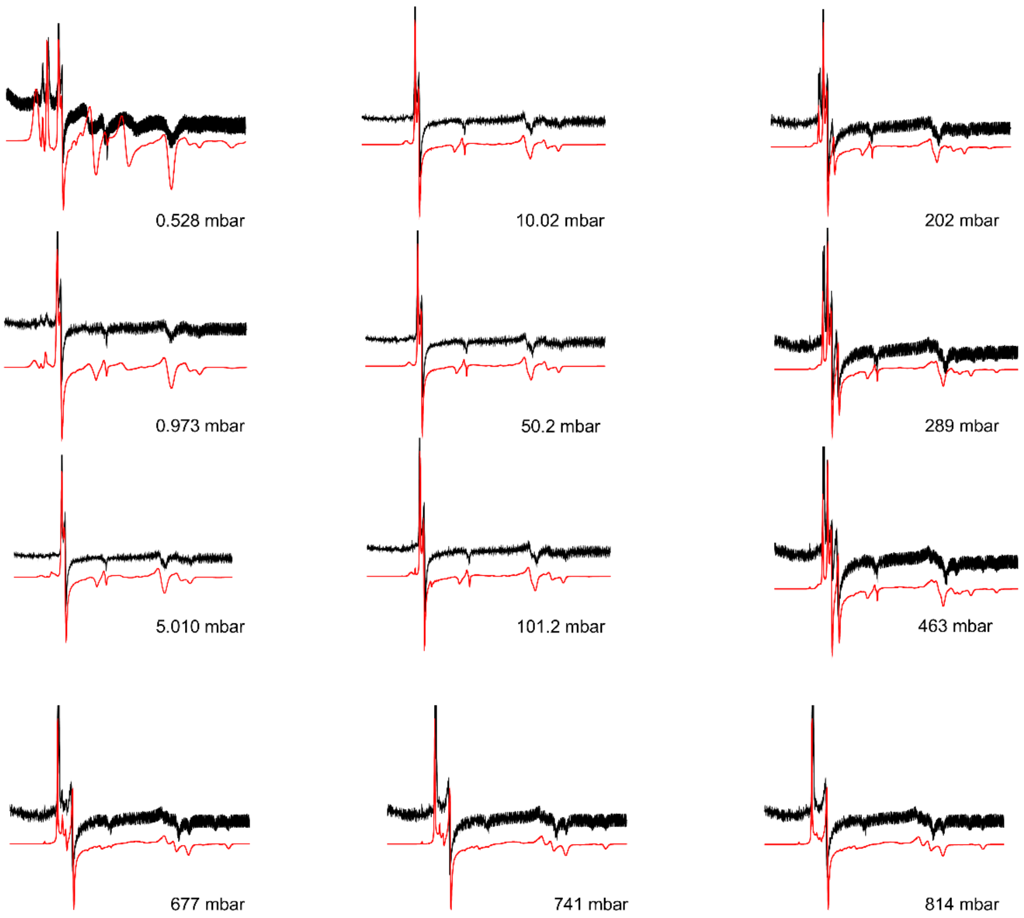

Figure S14. All experimental (black) and simulated (red) Cr^{3+} data for the D_2 adsorption on MIL-53 ($Al_{0.99}$ $Cr_{0.01}$) were recorded *in situ* at 23 K.

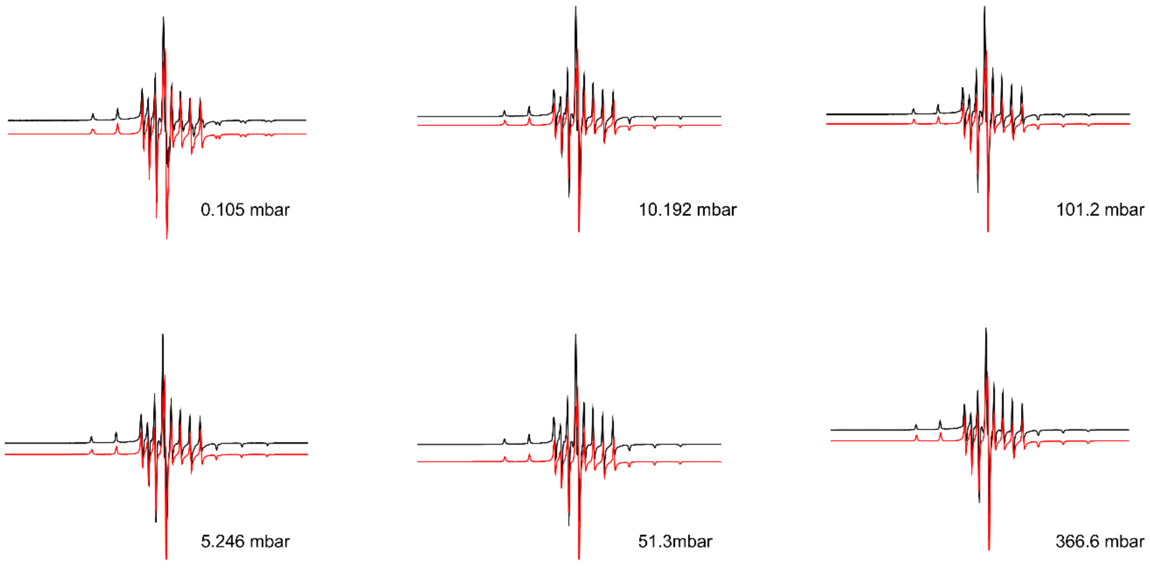

Figure S15. All experimental (black) and simulated (red) V^{4+} data for the D_2 adsorption on MIL-53 ($Al_{0.99}$ $V_{0.01}$) were recorded *in situ* at 21 K.

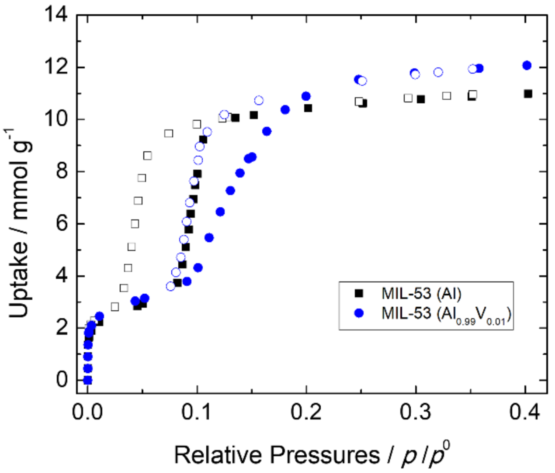

Figure S16. CO_2 sorption isotherm for MIL-53 (Al) and MIL-53 ($Al_{0.99}$ $V_{0.01}$) samples measured at 195 K.

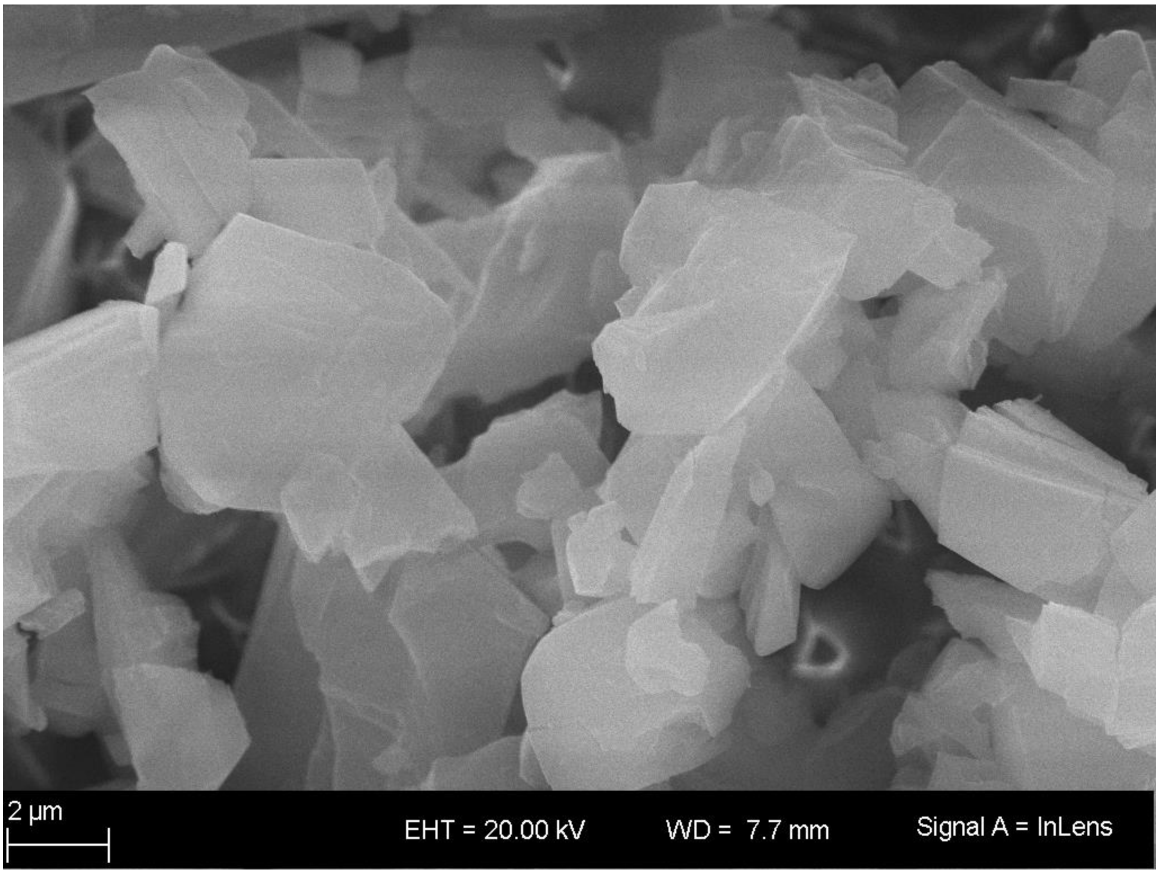

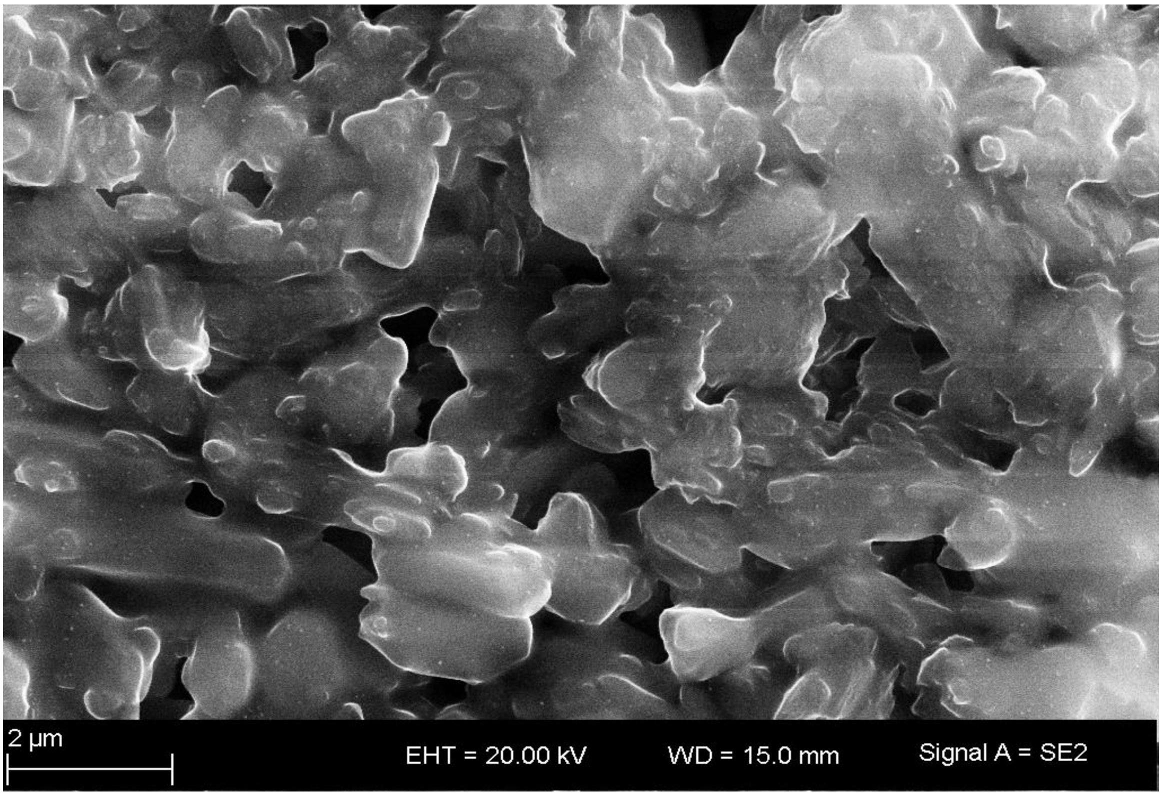

Figure S17. SEM image for MIL-53 ($Al_{0.99}$ $Cr_{0.01}$) sample (top) and MIL-53 ($Al_{0.99}$ $Cr_{0.01}$) (bottom).

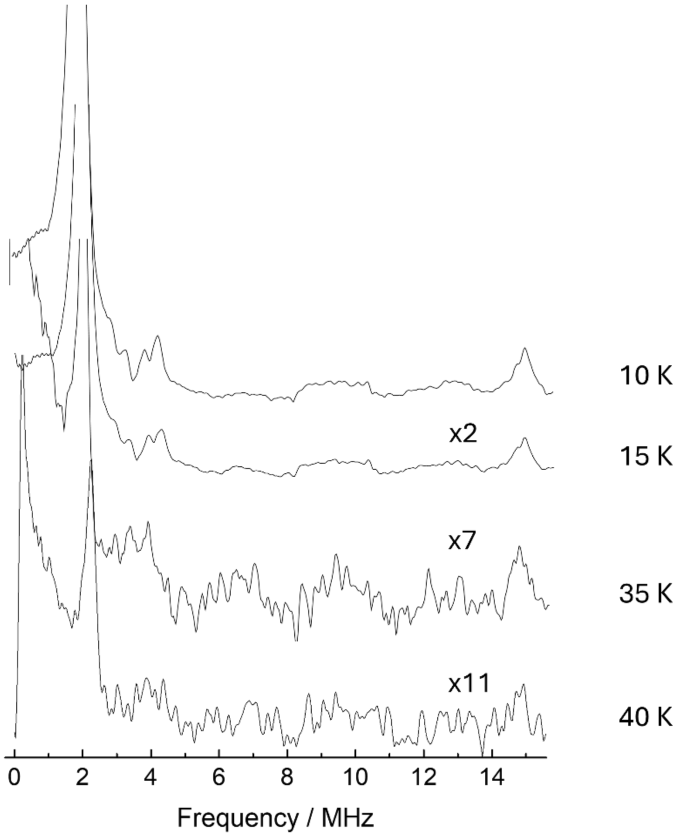

Figure S18. Frequency domain 3p ESEEM traces of D_2 desorption on MIL-53($Al_{0.99}V_{0.01}$) at several temperatures for comparison. Magnifications are implemented to closely evaluate the ^1H signal at ν_H = 14.74 MHz.

Table S2. Set of bonding properties (i.e. bond lengths and bond angles) for the Al^{3+} local structure within the MIL-53 (Al) crystal structures depending on the types of the pore phases.

Species	R_{ax}, Axial Al-O distance (nm)	R_{eq}, Equatorial Al-O distance (nm)	Δ_{ax}*, Degree of axial distortion	< OH-Al-OH (º)	< OH-Al-O (º)	< O-Al-O (º)
np	0.178609	0.173505<br>0.175458	-	180	86.24	94.52
lp	0.179293	0.175351	0.022	180	88.18	88.69
vlp	0.198895	0.186380	0.063	180	82.54	94.50

* Defined as $\Delta_{ax} = | R_{ax} - R_{eq} | / R_{ax}$

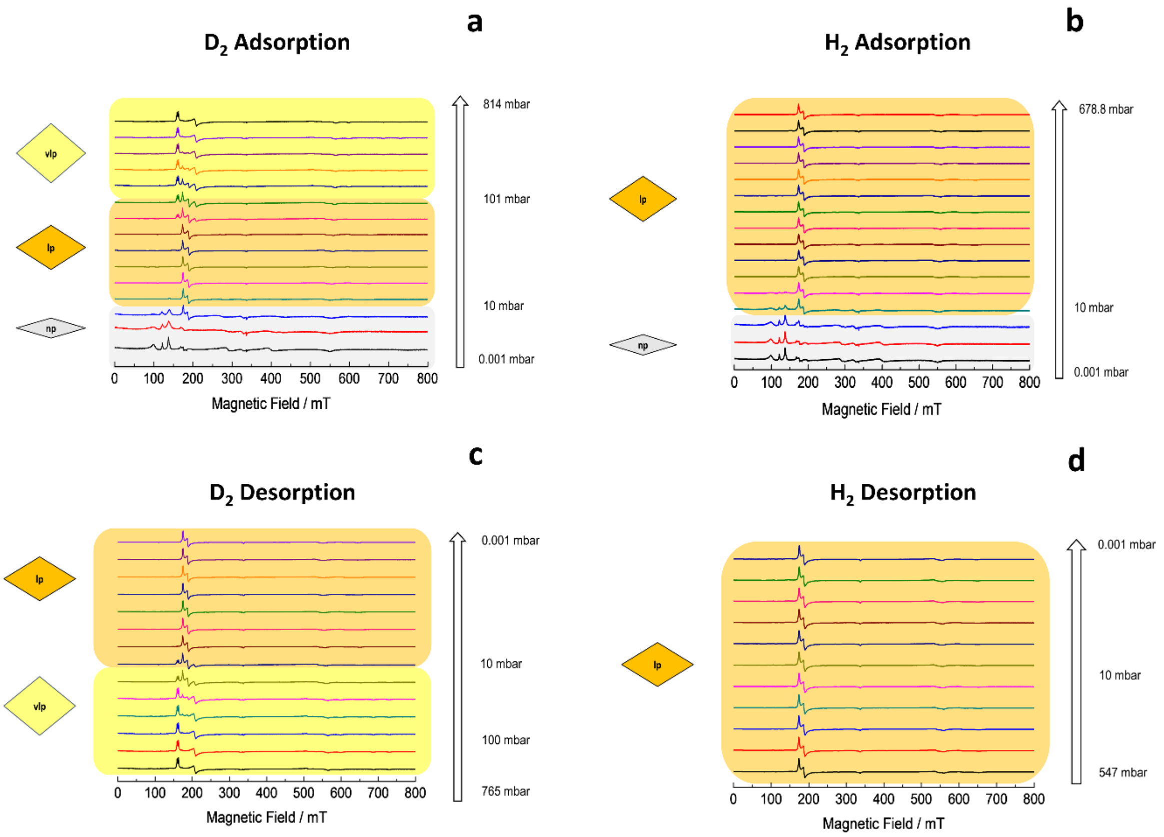

Figure S19. A complete set of *in situ* CW EPR spectra during D_2 and H_2 adsorption-desorption processes on MIL-53($Al_{0.99}Cr_{0.01}$) sample.

Author Contributions

M.F.L., S.C., V.B., P.S. and K.T. conducted the experiments and performed data analysis and interpretation. A.P., H.K., S.K. and M.H. reviewed the data and supported the data interpretation. A.P. and H.K. designed the project and acquired the funding. M.F.L., V.B., P.S. and A.P. wrote the manuscript with input from all authors.

References

1. Y. Liu, J. H. Her, A. Dailly, A. J. Ramirez-Cuesta, D. A. Neumann, C. M. Brown, *J. Am. Chem. Soc.* **2008**, *130*, 11813–11818.
2. M. Mendt, B. Jee, N. Stock, T. Ahnfeldt, M. Hartmann, D. Himsl, A. Pöppl, *J. Phys. Chem. C* **2010**, *114*, 19443–19451.
3. S. Stoll, A. Schweiger, *J. Magn. Reson.* **2006**, *178*, 42–55.
4. M. Mendt, B. Jee, D. Himsl, L. Moschkowitz, T. Ahnfeldt, N. Stock, M. Hartmann, A. Pöppl, *Appl. Magn. Reson.* **2014**, *45*, 269–285.
5. P. Höfer, *J. Magn. Reson. A* **1994**, *111*, 77–86.
6. I. Nevjestic, H. Depauw, K. Leus, V. Kalendra, I. Caretti, G. Jeschke, S. Van Doorslaer, F. Callens, P. Van Der Voort, H. Vrielinck, *ChemPhysChem* **2015**, *16*, 2968–2973.
7. Y. Jiang, J. Huang, S. Marx, W. Kleist, M. Hunger, A. Baiker, *J. Phys. Chem. Lett.* **2010**, *1*, 2886–2890.
8. I. F. Silvera, *Rev. Mod. Phys.* **1980**, *52*, 393.
9. D. M. Polyukhov, N. A. Kudriavykh, S. A. Gromilov, A. S. Kiryutin, A. S. Poryvaev, M. V. Fedin, *ACS Energy Lett.* **2022**, *7*, 4336–4341.
10. B. Jee, M. Hartmann, A. Pöppl, *Mol. Phys.* **2013**, *111*, 2950–2966.
11. S. Stoll, C. Calle, G. Mitrikas, A. Schweiger, *J. Magn. Reson.* **2005**, *177*, 93–101.
12. M. Mendt, P. Vervoorts, A. Schneemann, R. A. Fischer, A. Pöppl, *J. Phys. Chem. C* **2019**, *123*, 2940–2952.
